# Photocatalytic Reforming of Biomass: What Role Will the Technology Play in Future Energy Systems

**DOI:** 10.1007/s41061-022-00391-9

**Published:** 2022-06-18

**Authors:** Nathan Skillen, Helen Daly, Lan Lan, Meshal Aljohani, Christopher W. J. Murnaghan, Xiaolei Fan, Christopher Hardacre, Gary N. Sheldrake, Peter K. J. Robertson

**Affiliations:** 1 School of Chemistry and Chemical Engineering, Queens University Belfast, David Keir Building, Stranmillis Road, Belfast, BT9 5AL UK; 2 Department of Chemical Engineering, School of Engineering, The University of Manchester, Oxford Road, Manchester, M13 9P3AL UK

**Keywords:** Photocatalysis, Biomass, Hydrogen, Technology readiness level (TRL), Energy

## Abstract

Photocatalytic reforming of biomass has emerged as an area of significant interest within the last decade. The number of papers published in the literature has been steadily increasing with keywords such as ‘hydrogen’ and ‘visible’ becoming prominent research topics. There are likely two primary drivers behind this, the first of which is that biomass represents a more sustainable photocatalytic feedstock for reforming to value-added products and energy. The second is the transition towards achieving net zero emission targets, which has increased focus on the development of technologies that could play a role in future energy systems. Therefore, this review provides a perspective on not only the current state of the research but also a future outlook on the potential roadmap for photocatalytic reforming of biomass. Producing energy via photocatalytic biomass reforming is very desirable due to the ambient operating conditions and potential to utilise renewable energy (e.g., solar) with a wide variety of biomass resources. As both interest and development within this field continues to grow, however, there are challenges being identified that are paramount to further advancement. In reviewing both the literature and trajectory of the field, research priorities can be identified and utilised to facilitate fundamental research alongside whole systems evaluation. Moreover, this would underpin the enhancement of photocatalytic technology with a view towards improving the technology readiness level and promoting engagement between academia and industry.

## Introduction

### A Future Low-Carbon Energy Landscape

**Fig. 1:**
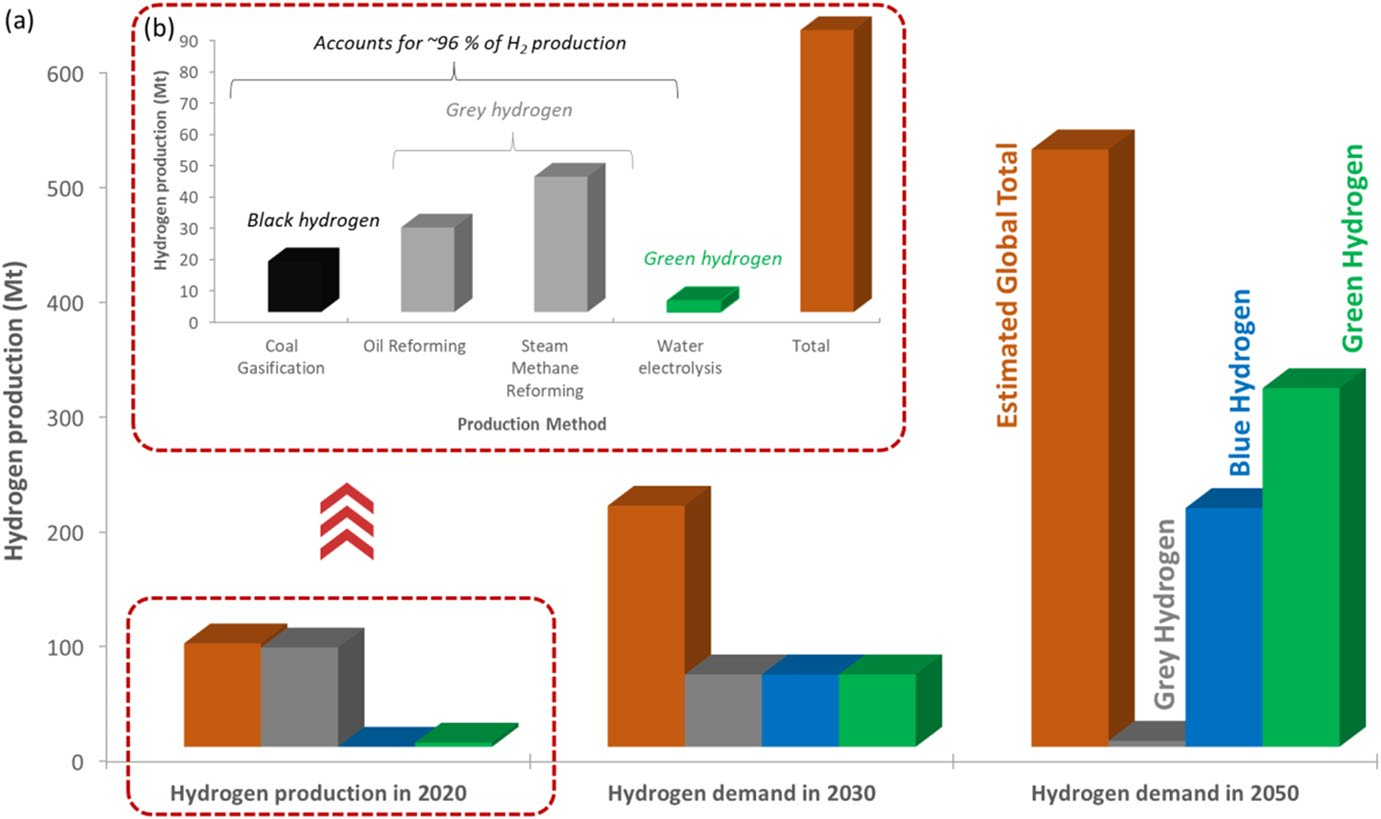
(**a**) Current (2020) and future global H_2_ production in relation to grey, blue and green H_2_ generation along with (**b**) a breakdown of production methods for H_2_ generation in 2020 including black, grey and green H_2_ [2–5]

**Fig. 2:**
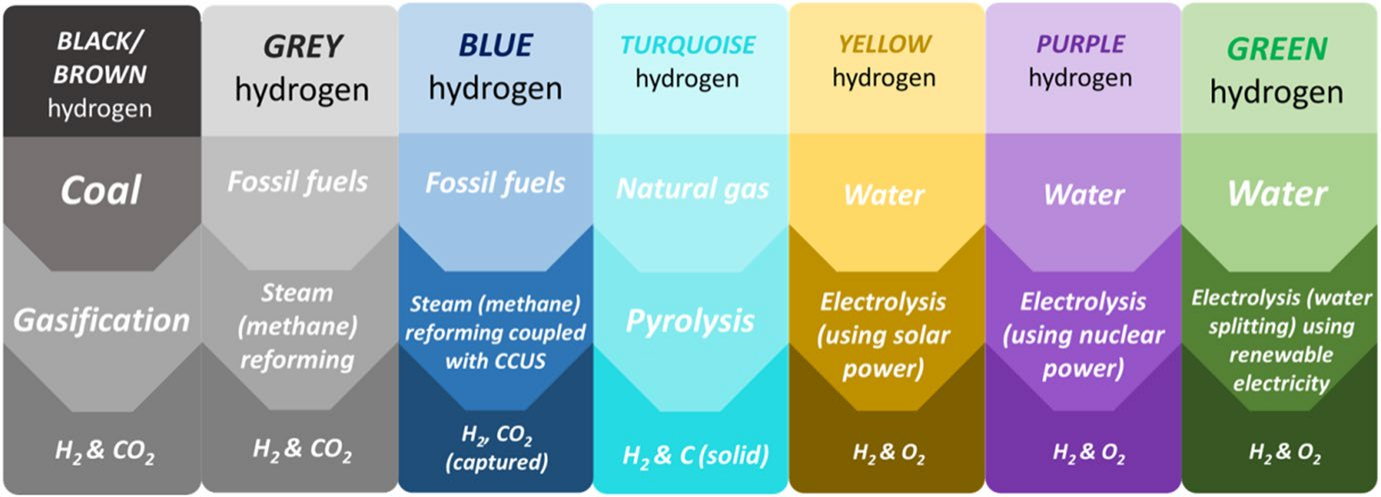
An overview of the H_2_ rainbow highlighting the feedstock, conversion process and output for each category

Countries around the globe have now set ambitious net zero emission (NZE) targets with the aim of facilitating a transition towards a green, low-carbon society. In 2019, the UK became one of the first major economies to pass laws that aligned with these targets by requiring all greenhouse gas emissions to be net zero (in comparison to levels in 1990) by 2050 [1, 2]. Achieving these targets is beyond simply stating that it will be ‘challenging’. An approach must be deployed that encourages engagement across disciplines, technologies, sectors, industries, and governments—all of which must be underpinned by national and international strategies, continuous dissemination, and appropriate financial support. A key component within the transition to a low-carbon future is the role that hydrogen (H_2_) will play in the global energy matrix. H_2_ is also a unique example that highlights the challenge associated with the transition to a greener society. H_2_ is already generated on a large global scale for use in industries such as refining, chemical synthesis and increasingly as an energy vector. The majority of that H_2_ (~ 96%) is currently generated via fossil fuels (e.g., steam methane reforming (SMR) or coal gasification); however, to achieve NZE targets, governments are shifting their efforts towards H_2_ produced via water electrolysis. This has given rise to categorising H_2_ based on feedstock and manufacturing routes and resulted in the development of the ‘hydrogen rainbow’. The term has now been adopted by several governments and industries to help illustrate their sustainable strategy for transitioning from grey H_2_ (fossil fuel derived) to green H_2_ (water electrolysis with renewable electricity). Figures 1 and 2 show the current manufacturing routes for global H_2_ in 2020 along with the predicted increase in 2030 and 2050, and an overview of the H_2_ rainbow, respectively.

To date, 17 countries have announced national H_2_ strategies, which emphasises the level of intent directed towards establishing a strong H_2_ economy. There is variation within each strategy; however, the majority have adopted a twin track approach wherein both green and blue H_2_ are identified as primary methods for delivering low-carbon H_2_ production (as indicated in the predicted demand for 2030 and 2050 in Fig. 1). In the UK specifically, a target capacity of 10 GW of low-carbon H_2_ is set for 2030, with an intermediate target of 1 GW by 2025 [6]. A more immediate target is to complete feasibility testing to allow the use of up to 20% H_2_ into the gas distribution grid for residential homes. According to the UK Government’s hydrogen strategy, achieving these targets is predicted to create a UK H_2_ economy that could be worth £900 million and generate 9000 high-quality jobs by 2030 [6]. In addition, modelling data suggest that 41 MtCO_2_e could be saved between 2023 and 2032 as a result [5]. While ambitious, these strategies also highlight the uncertainty that still exists around H_2_ deployment, specifically in relation to manufacturing routes, supply chains and the impact it could have on geopolitics. The International Energy Agency (IEA) predicted that to meet NZE by 2050, global H_2_ production must achieve two substantial objectives [3, 4]:

- **An increase in production:** from 90 Mt in 2020 to > 200 and > 500 Mt in 2030 and 2050, respectively
- **A shift in manufacturing routes:** from 95% grey H_2_ to 99% low-carbon production (e.g., a combination of blue and green H_2_)

The uncertainty that has arisen is aligned with the fact that green H_2_ production is still to be fully deployed at large scale as it currently accounts for only ~ 4% of global production. Furthermore, the cost of green H_2_ remains high at ~ 7 p/kWh (compared to < 4 p/kWh for SMR) and the current infrastructure in most countries would be unable to support a centralised distribution system [5]. It is, therefore, evident that alternative and complementary technologies will play a role in future energy systems. As a result, innovation and technology development will be crucial in contributing towards achieving low-carbon H_2_ production.

As a result of these challenges, increasing attention is being focussed on emerging technologies as alternative routes to H_2_ production which can support an expanding H_2_ economy at regional, national and international level. Along with research into thermolysis and redox chemistry [7], emerging technologies such as bioelectroylsis, biophotolysis and dark/photo-fermentation (as approaches to biohydrogen generation [8]) have gained significant attention in the literature. Beyond that, an area of particular interest is harnessing solar energy for the conversion of sustainable or waste feedstocks for H_2_ generation. Technologies such as artificial photosynthesis and photocatalysis are seen as highly desirable approaches due to their ability to valorise a range of feedstocks to both energy and value-added products.

### Photocatalytic Reforming of Biomass 

Photocatalytic reforming of biomass has grown significantly to become one of the most rapidly evolving applications in the field. The earliest report of photocatalysis being deployed for biomass conversion was by Kawai and Sakata in 1980, who demonstrated that generation of H_2_ from carbohydrates was occurring via photo-reforming [9]. In their work, the authors suggested that the carbon chains of compounds such as starch, sugar and cellulose were oxidised via reactions at the valence band (VB) which subsequently generated CO_2_ and protons. The protons were then reduced by electrons at the conduction band (CB) to produce H_2_. Despite this first paper being published alongside other early seminal work in photocatalysis [10–13], it is only within the last decade that a significant increase in publications has been observed. One of the primary drivers behind this has been the expansion of the bioenergy sector coupled with the focus on ensuring that sustainable and energy efficient processes are used for renewable energy generation. Furthermore, the ‘biorefinery concept’, which aims to achieve the production of both energy and value-added compounds from biomass, has highlighted that the sector could be reliant on using multiple conversion technologies [14–16]. Subsequently, this has led to an increase in the number of novel catalytic processes aimed at the valorisation of biomass. Interestingly, however, another key driver for photocatalysis has evolved from within the research field itself, especially in view of achieving sustainable H_2_ production [17]. While global renewable energy targets and sector growth have undoubtedly facilitated increased focus on photocatalysis for bioenergy, as a substrate, total biomass represents a photocatalytic feedstock that is potentially more economically viable than traditional ones such as alcohols, sugars, and acids. Water splitting remains a ‘holy grail’ for photocatalytic H_2_ production; however, with unfavourable thermodynamics (i.e., a large change in the Gibbs free energy with △*G*_o_ =  −238 kJ mol^−1^) and the limitation of back reactions (H_2_ and O_2_ forming H_2_O) [18], a more feasible approach is to deploy a sacrificial electron donor (SED) that can also supply protons. Previously, alcohols [19] and acids [20] have been used for this; however, biomass represents a potentially more sustainable and economically viable SED for photocatalytic systems. Moreover, the chemical and structural complexity of biomass can provide the added benefit of generating value-added compounds (bioproducts) during the process.

**Fig. 3:**
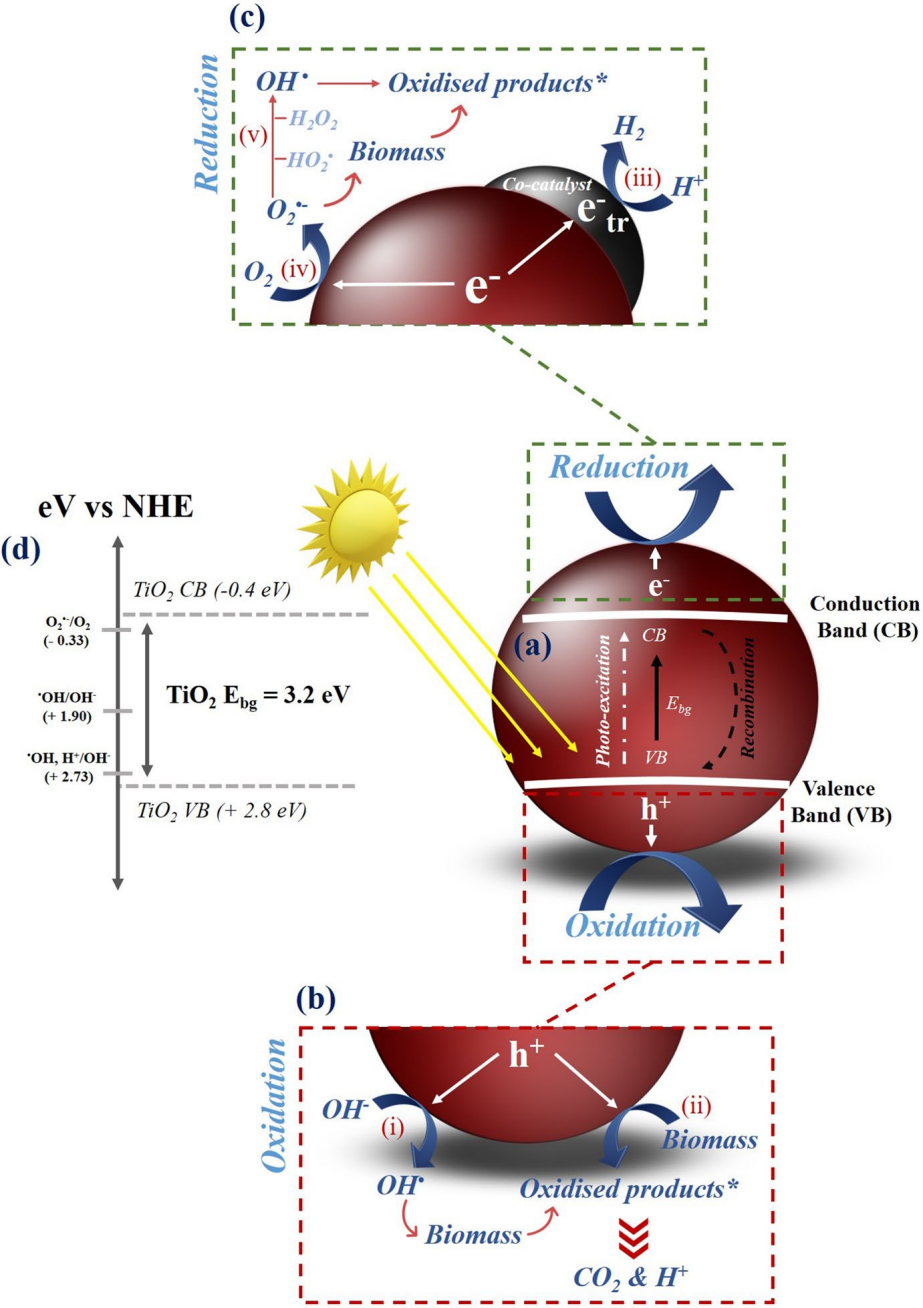
An overview of the photocatalytic mechanism for biomass reforming including (**a**) the electronic structure of TiO_2_ (as a model photocatalyst) and the processes of photo-excitation and recombination, the reactions which take place at (**b**) the valence band (including (i) radical attack and (ii) direct oxidation) (**c**) the conduction band (including (iii) H_2_ generation, (iv) superoxide generation and (v) H_2_O_2_ formation) and (**d**) the redox potentials associated with the generation of reactive oxygen species. Please note * refers to a range of oxidised products which vary depending on the chemical structure of the biomass substrate. Adapted from [34] with kind permission from Elsevier

The reactions which occur during photocatalysis have been extensively reviewed in a number of excellent papers in the literature [21–28] with the core mechanism often remaining the same, irrespective of application. Photocatalysis is a light driven chemical process which utilises oxidation and reduction reactions via the generation of an electron–hole pair. The photocatalytic mechanism with respect to biomass as the reaction substrate is shown in Fig. 3, which utilises TiO_2_ as a model photocatalyst. Provided photon energy equal to or greater than the energy band gap (*E*_bg_) of the absorbing material (i.e., the photocatalyst) is provided, ground state electrons can be promoted to higher energy levels and in doing so create an electron–hole pair (Fig. 3a). The migration of the electron–hole pair to the catalyst surface facilitates redox reactions with absorbed species. Using an aerated aqueous solution containing biomass as an example, the photogenerated charge carriers are capable of reacting with H_2_O and dissolved O_2_ on or near the surface to generate reactive species, e.g. a hydroxyl radical (∙OH), a superoxide anion (O_2_^•−^) and H_2_O_2_ (Fig. 3b, c), providing the redox potential of the reactions are met (Fig. 3d).

In biomass photocatalysis, the role of reactive oxygen species (ROS) and the surface interaction become crucial considerations. Biomass substrates can, in theory, undergo oxidation via direct (Fig. 3b, i—reaction with the h_VB_^+^) and indirect (Fig. 3b, ii—reaction with the ∙OH) mechanisms to form reaction intermediates. In principle, the O_2_^•−^ generated from the electron scavenging at the CB can also react with biomass to form reaction intermediates (Fig. 3c, iv). While it is yet to be reported in the literature, the H_2_O_2_ pathway (Fig. 3c, v) may also contribute towards generating intermediates via ∙OH attack. These intermediates can vary significantly depending on the composition of the starting substrate and are also subject to complex reaction pathways, which are yet to be fully elucidated in the literature. If cellulose is considered as a standard biomass model compound, reaction intermediates (denoted in Fig. 3 as *oxidised products) can include sugars such as cellobiose, glucose, arabinose and erythrose, platform chemicals such as HMF, and organic acids such as acetic and formic acid [29–31]. Although the mechanism is not fully understood, it is generally accepted that biomass can act as an effective SED for photocatalytic systems. The irreversible oxidation which occurs subsequently leads to a supply of both electrons and protons, with the latter facilitating the generation of H_2_ at the CB (Fig. 3c, iii). To enhance H_2_ production, a co-catalyst (e.g., Pt, Ni, Au or Pd [20, 32, 33]) is typically used as a reaction centre to trap electrons (e^−^_tr_) with operation under an inert atmosphere (e.g., N_2_ purged) removing competition from O_2_ for scavenging the electron.

The growth of this area is primarily reflected in the literature, with the number of papers on photocatalytic biomass conversion increasing rapidly over the past decade. Moreover, research trends also highlight the shift from early work which focused on simple carbohydrate models (i.e., sugars) [35, 36] to more recent work which utilises raw biomass (i.e., lignocellulosic material) for H_2_ [32, 37], with examples also demonstrating activity under solar irradiation [29, 38]. While these reports represent the advancement of the technology, they also highlight key challenges which must be addressed. The development and deployment of photocatalysis as a technique for biomass reforming is underpinned by not just overcoming these challenges, but by identifying a roadmap that will enhance the technology readiness with a view towards contributing to achieving net zero targets.

This article will explore these points in detail and provide a perspective on both the current state of the research and predicted future impact. A critical review is provided that evaluates current progress in the field which will be structured on product formation—specifically H_2_ and value-added compounds. Following this, the article will focus on providing the reader with an outlook on future developments based on the previous critical evaluation. This includes identifying and categorising challenges and assessing the technology readiness level (TRL) of photocatalytic reforming of biomass before proposing a roadmap which outlines a strategy to facilitate future advancement of the technology. Biomass conversion is an emerging topic in photocatalytic research; however, to date, previous articles are either research-based or reviews of published research. For the first time in this field, this article evaluates the literature but also addresses the technology horizon and feasibility of the process for real-world deployment.

## Current State of the Research

### Photocatalytic H_2_ Generation

**Fig. 4:**
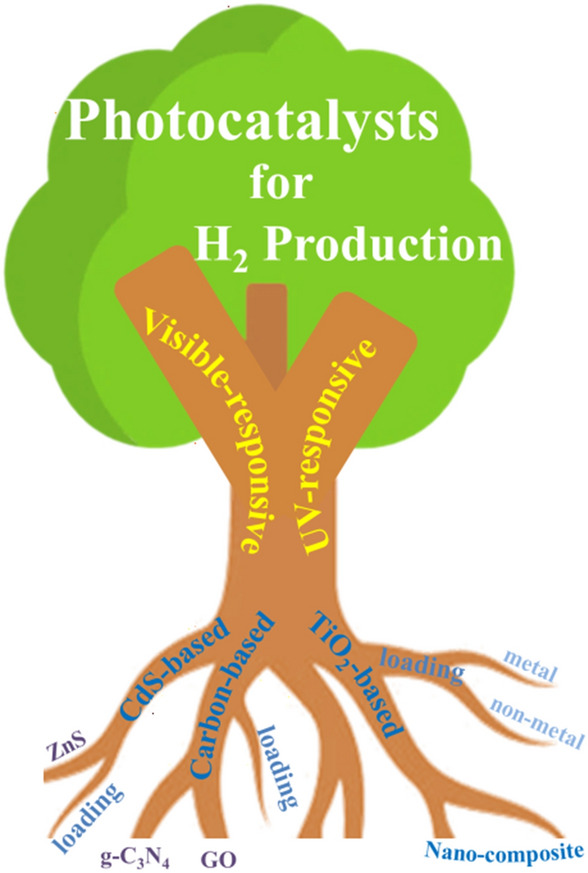
Illustration overview of common photocatalysts and their synthesis strategies for H_2_ production from photocatalytic reforming of lignocellulosic materials

Research on photocatalytic H_2_ generation from biomass feedstocks, including cellulosic materials (i.e., cellulose, hemicellulose and lignin) and waste streams (e.g., wood, grass, and waste papers) are critically reviewed in this section. To accompany this, a comprehensive overview of the state-of-the-art information of photo-reforming of lignocellulosic materials is shown in Table 1. Unless otherwise stated, the generation of H_2_ from such photocatalytic systems is assumed to proceed via the mechanism detailed in the previous section and in Fig. 3. The H_2_ production (µmol) collected from the current literature was normalised by the amount of catalyst used (*g*_cat_) and irradiation time (*h*) to obtain a H_2_ production rate (*r*H_2_, µmol h^−1^g_cat_^−1^), which was used for comparing the activity of photo-reforming of lignocellulosic materials over different catalysts or in different systems. Where appropriate, efficiencies (%) are also reported as either apparent quantum yields (AQY) or solar-to-hydrogen (STH). TiO_2_-based and non-TiO_2_-based (e.g., CdS-, ZnS-, CN_x_-, carbon-based) catalysts were compared for their synthesis methods (as illustrated in Fig. 4) and activity of H_2_ production (*r*H_2_) from photo-reforming of lignocellulosic materials. Photocatalytic reaction systems under the irradiation of solar simulators and natural sunlight are also reviewed based on their choices of photocatalysts, *r*H_2_ and system design.

**Table 1:** The state-of-the-art data for the photocatalytic reforming of lignocellulosic materials

Photocatalyst	Substrate	Reaction conditions					H_2_ production rate	AQY/wavelength^d^	Other products	Refs
		Light source	*P*/*I*	*T* (°C)	Concentration (g L^−1^)^b^		(µmol h^−1^g_cat_^−1^)^c^	(%)/(nm)		
			(W)/ (mW cm^−2^)^a^		Catalyst	Substance/other solvent				
Pt/RuO_2_/TiO_2_	Cellulose	Xe	500/–	25	7.5	3.0	12	0.3/380	CO_2_, CH_3_OH, C_2_H_5_OH	[9]
Pt/TiO_2_	Cellulose	Xe	500/–	–	10	3.3	13	–	–	[63]
Pt/TiO_2_	Cellulose	Xe	150/–	60	0.8	1.0	232	–	–	[32]
Pt/TiO_2_	Cellulose	UV	4 × 15 W/–	–	2.0	6.7	225	–	Glucose, HMF	[46]
Pt/TiO_2_	Cellulose	Simulator	–/25	–	2.0	6.7	185	–	–	[46]
Pt/TiO_2_	Cellulose	Sunlight	–/45 (2.5)^e^	–	2.0	6.7	196	–	–	[46]
Pt/TiO_2_	Cellulose	UV	250/–	130	2.0	0.1/0.6 M sulfuric acid	752	–	HMF	[45]
mPt/TiO_2_	Cellulose	Xe	300/–	40	1.5	1.0% w/v^f^	600	9.6/365	–	[40]
Pt/TiO_2_	Cellulose	Xe	300/–	40	1.5	1.0% w/v /HCl	900	14.5/365	–	[41]
Pt/TiO_2_	Cellulose I	UV LED	13.2/5	28.5	0.75	4	40	3.9/–	–	[42]
Pt/TiO_2_	Cellulose II	UV LED	13.2/5	28.5	0.75	4	104	9.4/–	–	[42]
Pt/TiO_2_	Cellulose I	UV	16/	40	0.75	1	107	25.9/365	CO_2_	[64]
Pt/TiO_2_	Cellulose II	UV	16/	40	0.75	1	177	42.8/365	CO_2_	[64]
Pt/TiO_2_	Cellulose	UV	16/–	40	0.75	1	133	–	CO_2_	[43]
Pt/TiO_2_	Cellulose	Xe	300/–	–	0.4	4	275	1.9/380	Lactic acid, arabinose, glucose, mannose, galacturonic acid	[47]
Pd/TiO_2_	Cellulose	UV–Visible lamp	150/–	–	1	3.3	1456	–	–	[44]
Ni-S/TiO_2_	Cellulose	Xe	500/400	80	0.2	10.0	3020	–	Arabinose, galactose, glucose, xylose, formic acid	[48]
Au/HYT ^g^	Cellulose	Visible	–/0.5	140	0.1	0.1/EMIMCl^h^	–	–	Glucose, HMF	[65]
TiO_2_ film	Cellulose	UV	–/–	–	9 coatings	100/ZnCl_2_	–	–	HMF	[66]
Cellulose@Pt/TiO_2_	Cellulose	UV	250/–	40	0.3	–	933	–	Glucose, formic acid	[29]
NiP-^NCN^CN_x_^i^	Cellulose	Simulator	–/100	25	5.0 mg	10.0 M/KPi^j^	1690	–	–	[52]
NiS/CdS	Cellulose	Xe lamp	300/–	–	1	10	53	–	–	[50]
CdS/CdO_x_ QDs^k^	Cellulose	Simulator	–/100	25	0.5 µM	50/10 M, KOH	2300	1.2/430	–	[38]
Pt/SNGODs ^l^	Cellulose	Simulator	–/100	–	0.8	4/10 M, NaOH	431	23.3/420	HCOO–, C6 to C1	[57]
CdS/CdO_x_ QDs	Hemicellulose^m^	Simulator	–/100	25	0.5 µM	0.25/10 M, KOH	2000	1.2/430	–	[38]
NiP-.^NCN^CN_x_	Hemicellulose^n^	Simulator	–/100	25	1.6	33.3/4.3 M, KPi	137	–	–	[52]
Pt/g-C_3_N_4_	Hemicellulose	Xe	300/100	5	5	5/pH = 10	60	–		[53]
Pt/TiO_2_	Lignin	Xe	500/–	–	10	3.3	4	–	–	[67]
TiO_2_@NiO	Lignin^o^	Xe	300/	60	0.5	4/1 M, NaOH	78	–	CH_4_, fatty, palmitic, stearic and butanedioic acids	[49]
C,N,S-doped ZnO/ZnS	Lignin^p^	Xe	300/–	–	0.5	0.1	6430	15.1/–	1-phenyl-3-buten-1-ol	[68]
CdS/CdO_x_ QDs	Lignin	Simulator	–/100	25	0.5 µM	0.25/10 M, KOH	260	1.2/430	–	[38]
NiP-.^NCN^CN_x_	Lignin	Simulator	–/100	25	1.6	0.16/4.3 M, KPi	40.8	–	–	[52]
Pt-.^NCN^CN_x_	Lignin	Simulator	–/100	25	1.6	0.16/10 M, KOH	14.5	–	–	[52]
Pt/g-C_3_N_4_	Lignin	Xe	300/100	5	5	5/pH = 10	20.75	–		[53]
NiS/CdS	Lignin	Xe	300/	25	1	0.1/lactic acid	147.6	44.9/–λ ≥ 400 nm		[51]
Pt/TiO_2_	Poplar wood	Xe	300/–	–	0.4	4	26	–	–	[47]
Pt/TiO_2_	Pinewood	Simulator	–/200	160	30.0	–/HCl	3757	1.1^p^	–	[58]
Pt/TiO_2_	Fescue grass	Xe	300/–	60	0.8	1.8	68	–	–	[32]
Pt/TiO_2_	Rice husk	UV	–/–	–	2	6.7	100	–	–	[46]
Pt/TiO_2_	Alfalfa stem	UV	–/–	–	2	6.7	100	–	–	[46]
Pt/TiO_2_	Paper pulp		UV	250/-	130	0.5	10 /H_2_SO_4_	1320	–	[45]
CdS/CdO_x_ QDs	Grass	Simulator	–/100	–	0.5 M	50.0/10 M, KOH	900	–	–	[38]
CdS/CdO_x_ QDs	Printer paper	Simulator	–/100	–	0.5 M	50.0/10 M, KOH	1050	–	–	[38]
CdS/CdO_x_ QDs	Cardboard	Simulator	–/100	–	0.5 M	50.0/10 M, KOH	680	–	–	[38]
CdS/CdO_x_ QDs	Newspaper	Simulator	–/100	–	0.5 M	50.0/10 M, KOH	320	–	–	[38]
CdS/CdO_x_ QDs	Wooden branch	Simulator	–/100	–	0.5 M	50.0/10 M, KOH	5350	–	–	[38]
CdS/CdO_x_ QDs	Bagasse	Simulator	–/100	–	0.5 M	50.0/10 M, KOH	250	–	–	[38]
CdS/CdO_x_ QDs	Sawdust	Simulator	–/100	–	0.5 M	50.0/10 M, KOH	720	–	–	[38]
NiP-.^NCN^CN_x_	Sawdust	Simulator	–/100	25	1.6	0.16/4.3 M, KPi	202	–	–	[52]
NiP-.^NCN^CN_x_	Paper	Simulator	–/100	25	1.6	0.16/4.3 M, KPi	42.7	–	–	[52]
NiP-.^NCN^CN_x_	Cardboard	Simulator	–/100	25	1.6	0.16/4.3 M, KPi	46.9	–	–	[52]
NiP-.^NCN^CN_x_	Bagasse	Simulator	–/100	25	1.6	0.16/4.3 M, KPi	34.8	–	–	[52]
NiP-.^NCN^CN_x_	Wooden branch	Simulator	–/100	25	1.6	0.16/4.3 M, KPi	35.7	–	–	[52]

^a^Light intensity was shown in two units in the literature, i.e., in radiant flux power (*P*), or in irradiance (*I*)

^b^Default unit is g L^−1^ if there is no noted unit following the value. Various units were noted as they were shown in different ways in the original literature

^c^Solvents (other than water) were used to dissolve the substrates or adjust the pH of the system

^d^The production rate of H_2_ is the amount of H_2_ produced per hour (µmol h^−1^) normalised by the amount of catalyst (g) used in the studies

^e^AQY is the abbreviation for apparent quantum yield, which is calculated by the ratio of the molar mass of transferred electrons to the molar mass of incident photons

^f^The average solar power of the natural sunlight used in this study was 45 mW cm^−2^ in the visible range and 2.5 mW cm^−2^ in the UV range

^g^w/v represents the weight/volume percentage concentration

^h^HYT is the abbreviation of TiO_2_ nanofibres supported H-form Y-zeolites (HY) catalyst

^i^EMIMCl is 1-ethyl-3-methylimidazolium chloride ionic liquid solution which is used to dissolve cellulose as a pre-treatment in this study

^j^NiP-^NCN^CN_x_ is the bulk cyanamide-functionalised carbon nitride (^NCN^CN_x_) with molecular Ni bis(diphosphine) (NiP) as the co-catalyst

^k^KPi is a potassium phosphate solution used in this study

^l^QDs is the abbreviation of quantum dots, and (Co(BF_4_))_2_ is used as the co-catalyst in this study

^m^SNGODs is the abbreviation of the S- and N-doped grapheme oxide dots (SNGODs)

^n^The hemicellulose used in this study is xylan from beech wood

^o^The hemicellulose used in this study is xylan

^p^Kraft lignin was used as the lignin source in this study

^q^Sodium salt lignisulfonate was used as the lignin source in this study

^r^The solar-to-H_2_ efficiency is calculated by the ratio of calculated energy corresponding to H_2_ volume generated by taking into account the energy content of H_2_ (142 MJ/kg) to the energy supplied during photocatalysis at different irradiation intensities

#### Photocatalysts for H_2_ Production

TiO_2_ is frequently used as a photocatalyst due to its suitable band gap energy (around 3.2 eV) under UV irradiation, and due to favourable characteristics such as its cost effectiveness, chemical stability and relatively low toxicity. While TiO_2_ has previously been reported as non-toxic, it is worth noting that as of 2021, the European Union classified TiO_2_ as a suspected carcinogen (category 2) by inhalation. The activity of bare TiO_2_ for H_2_ production from photocatalytic reforming of lignocellulosic materials, however, is relatively low, mainly as a result of the rapid recombination of photogenerated electrons (e_CB_^−^) and holes (h_VB_^+^) [39]. In the initial work by Kawai and Sakata, improvements on the efficiency of separating e_CB_^−^ and h_VB_^+^ was achieved by physically mixing and grinding the powders of RuO_2_, TiO_2_ and Pt together [9]. This mixed catalyst was then suspended, with cellulose, in H_2_O for H_2_ production under the irradiation of a Xe arc lamp. This first study showed the feasibility of cellulose as a SED in photo-reforming wherein H_2_ production of 12 µmol h^−1^ g_cat_^−1^ was achieved. The efficiency and yields of H_2_ production with cellulose as an SED are compared in Table 1 with the enhanced performance mainly achieved through catalyst synthesis strategies such as (i) optimisation of metal or non-metal co-catalyst loading on the TiO_2_ support, and (ii) fabricating novel TiO_2_-based composites.

Wet impregnation [32, 40–44] and photodeposition [45–47] methods are frequently used for loading metals (e.g., Pt, Au or Pd) as the co-catalyst onto a TiO_2_ support, with Pt the most commonly studied. As shown in Table 1, the *r*H_2_ of these noble metal loaded TiO_2_ catalysts for photo-reforming of lignocellulosic materials are in the range of 100–1000 µmol h^−1^ g_cat_^−1^. While the activity can obviously be enhanced by loading noble metals, the high costs of such materials have implications on the commercial and economic viability at scale. Non-noble metal (or non-metal) species have also been introduced onto TiO_2_ supports for H_2_ generation via photo-reforming of cellulose. Hao et al*.* [48] successfully prepared a Ni-S/TiO_2_ catalyst on which sulfate (SO_4_^2−^) and nickel sulfide (Ni_x_S_y_) were chemisorbed onto the TiO_2_. The *r*H_2_ (3020 µmol h^−1^ g_cat_^−1^) was significantly improved, by 96 times, after loading both Ni_x_S_y_ and SO_4_^2−^ on TiO_2_. This enhancement could be due to the synergistic role of both Ni_x_S_y_ and SO_4_^2−^; the Ni_x_S_y_ served as both electron trap and co-catalyst for H_2_ evolution, while binding SO_4_^2−^ generates acidic sites in the catalyst which can facilitate insoluble cellulose hydrolysis to soluble glucose, which is more efficient for quenching photogenerated holes and providing protons.

Novel TiO_2_-based composite structures have also been developed for H_2_ generation via the photo-reforming of lignocellulosic materials. A TiO_2_@NiO core–shell composite was prepared by mixing Ti(OC_3_H_7_)_4_ and Ni(CH_3_COO)_2_ in acetic acid with hydrothermal (180 °C for 12 h) and calcination (600 °C for 2 h) treatments [49]. This nanocomposite was capable of generating H_2_ (*r*H_2_ = 78 µmol h^−1^ g_cat_^−1^) from lignin photo-reforming due to the synergistic effects between ultrafine nanoparticles, high crystallinity, core–shell structure with close contact and a n-p heterojunction which facilitated the efficient separation of the photogenerated electron–hole pair. Additionally, Zhang et al. prepared a cellulose-immobilised Pt/TiO_2_ (cellulose@Pt/TiO_2_) composite by filtering a mixed colloidal suspension of cellulose and platinised TiO_2_ [29]. The study also compared a physically mixed cellulose and Pt/TiO_2_ (cellulose + Pt/TiO_2_) sample with the composite cellulose@Pt/TiO_2_ material (with both containing the same amounts of cellulose) for their activity of producing H_2_ from cellulose photo-reforming. The *r*H_2_ was approximately 933 µmol h^−1^ g_cat_^−1^ over the cellulose@Pt/TiO_2_ while the value was negligible over the cellulose + Pt/TiO_2_ sample, which indicated the importance of interaction between cellulose and the Pt/TiO_2_ for efficient photocatalytic H_2_ production. This could be due to the efficient transfer of photogenerated charge carriers (e_CB_^−^ and h_VB_^+^) and ROS (i.e., ∙OH) within the system.

Visible light-responsive photocatalysts such as sulfides (*E*_bg_ = ⁓2.4 eV CdS [38, 50, 51]) and carbon nanomaterials (*E*_bg_ = ⁓2.7 eV for graphitic carbon nitride [52, 53]) have also been developed for H_2_ generation from photo-reforming of lignocellulosic substrates. 1D CdS nanowires (NWs) were prepared and loaded with NiS_2_ by a two-step hydrothermal method. This catalyst showed visible light (*λ* ≥ 400 nm) reactivity towards H_2_ generation from photo-reforming of cellulose [50] and lignin [51] with a *r*H_2_ = 53 and 147.6 µmol h^−1^ g_cat_^−1^, respectively, where lactic acid was coexistent with lignin as a hole scavenger [47], whereas cellulose was used as the single hole scavenger [46] over NiS_2_/CdS catalysts. The NiS_2_ loading on CdS NWs was reported to enhance light absorption and charge transfer, which resulted in the visible light activity in producing H_2_. In addition to CdS NWs, CdS quantum dots were also reported to achieve high rates of H_2_ evolution from photo-reforming of lignocellulosic materials (*r*H_2_ = 2300, 2000, and 260 µmol h^−1^ g_cat_^−1^ for cellulose, hemicellulose, and lignin respectively) and even raw biomass (*r*H_2_ = 900 and 720 µmol h^−1^ g_cat_^−1^ for grass and sawdust respectively) in highly basic conditions (10 mol L^−1^ KOH) [38]. The high rates of H_2_ production for this catalyst could be due to the coverage of CdO_x_ on CdS which promotes the binding between the substrates and photocatalyst. This resulted in effective H^+^ transfer and weakening of C–C bonds, which in turn led to oxidation of the substrates and H_2_ production. Consideration of the overall sustainability and environmental impact of the production of photocatalytic H_2_, however, would require inclusion of the catalyst synthesis/precursors. Therefore, use of more environmentally benign metals in the preparation of active photocatalysts would be required.

Carbon nanomaterials are normally non-toxic with a suitable band gap (e.g., 2.7 eV for graphitic carbon nitride, g-C_3_N_4_ [54]) for absorbing visible light irradiation due to their 2D conjugated π-system. Activated carbon nitride (^NCN^CN_x_) was synthesised by an ultrasonication approach of breaking down aggregates of carbon nitride [52]. Cellulose, lignin, and raw biomass substrates were photo-reformed to H_2_ over ^NCN^CN_x_ (NiP as the co-catalyst) under the irradiation of a solar simulator, which demonstrates nontoxic, noble-metal free, and visible-light-promoted photo-reforming of lignocellulosic materials. Rao et al. [53] reported a monolayer g-C_3_N_4_ prepared by nitrogen-protected ball-milling of bulk g-C_3_N_4_ in water, which showed activity towards photo-reforming of lignin and hemicellulose to H_2_ driven by visible light; the *r*H_2_ was 60 (hemicellulose) and 20.8 (lignin) µmol h^−1^g_cat_^−1^, with Pt as the co-catalyst. For 2D layered carbon-based materials, a thinner layered structure could result in larger surface area and more active sites, which would enhance activity for producing H_2_ from photocatalytic reaction [54]. An increased band gap energy, however, could also be found with a thinner layer of this material due to quantum confinement effects by shifting the VB and CB in opposite directions [55]. Therefore, modification of g-C_3_N_4_ still needs to be developed to achieve enhanced performance in the production of H_2_ from the photo-reforming of lignocellulosic materials under visible-light irradiation. In addition, in the development of photocatalysts for photo-reforming of lignocellulosic substrates, consideration must be given to the nature of the feedstock used when comparing the activities of photocatalysts. The *r*H_2_, particularly for lignin resources can vary significantly with reaction conditions (i.e., acid/alkaline/neutral solutions and concentration of substrate) as well as the solubility of the lignin resource (for example lignosulfonate, organosolv or kraft lignin compared to raw biomass). One of the highest *r*H_2_ reported for lignin is for a C-, N-, and S-doped mixed phase ZnO and ZnS photocatalyst (C, N, S-ZnO/ZnS), prepared via calcination of bis-thiourea zinc acetate mixture, which demonstrated an *r*H_2_ of 6430 µmol h^−1^ g_cat_^−1^ from photo-reforming of sodium lignosulfonate salt under visible-light irradiation [50]. In comparison, however, for raw biomass the *r*H_2_ is typically below 1000 µmol h^−1^ g_cat_^−1^, for both UV and visible activation (Table 1).

#### Solar Irradiation

Sunlight is a sustainable source of energy; therefore, the efficient use or transfer of solar energy has been regarded as a promising and renewable approach to replace more traditional energy sources (e.g., fossil fuels) [56]. Photo-reforming of lignocellulosic materials for H_2_ production under natural sunlight represents an ideal method to transfer the solar energy to low carbon H_2_. Within the spectrum of natural sunlight, UV light represents only a small portion (~ 3–5% on an energy basis [39]) with the rest of the solar spectrum comprising wavelengths in the visible and infrared region. Therefore, in order to utilise natural sunlight efficiently, the development of visible light-responsive photocatalysts and the design of photoreactors which can fully harvest the irradiation of natural sunlight needs to be achieved.

Several studies reported in the literature have used solar simulators to mimic natural sunlight by adding an air mass filter (AM1.5G) to adjust the spectrum of a Xe arc lamp to the standard solar irradiation at ground level, with an approximate light intensity of 100 mW cm^−2^ [38, 52, 57, 58]. Such systems have been reported for the photo-reforming of biomass (i.e., cellulose, lignin, and raw biomass) over visible light responsive catalysts such as CdS/CdO_x_ quantum dots [38] and NiP/^NCN^CN_x_ [52]. H_2_ was produced over both catalysts and with various substrates including cellulose, hemicellulose, lignin, and raw biomass (shown in Table 1). Additionally, the effect of the illumination wavelength of the solar simulator on the efficiency of producing H_2_ was illustrated by an apparent quantum yield (AQY) in the photo-reforming of cellulose [57]. The results showed that both AQY and absorbance decreased with the irradiation wavelength, i.e., the AQY of 23.3% was achieved at 420 nm, and it decreased to 3.4% at 550 nm. The authors stated that this trend could be due to the low probability of the charge excitation under long-wavelength illumination.

Reports on using natural (and direct) sunlight as the irradiation source for producing H_2_ from photo-reforming of biomass are limited [29, 46]. A system containing an aqueous cellulose suspension with a Pt/TiO_2_ catalyst was irradiated under natural solar light (Pavia, 45°11′N, 9°09′E, July 2013, temperature 29–32 °C) for monitoring H_2_ production [46]. A *r*H_2_ of 196 µmol h^−1^g_cat_^−1^ was observed under natural sunlight, which was comparable to the value obtained from a solar simulator, but lower than that from a UV-A system (as shown in the Table 1). The composition and intensity of irradiating light, however, were different between the solar simulator and natural sunlight systems. The UV light was removed by a UV filter for the solar simulator system (25 mW cm^−2^), while UV light was present in the natural sunlight system, which was reported to have an average solar power of 2.5 mW cm^−2^ for the UV range and 45 mW cm^−2^ for the visible range. Simultaneous production of H_2_ and liquid phase compounds (e.g., sugars and organic acids) was monitored under direct solar irradiation (St Andrews, Scotland, UK, July, temperature 16 °C) in an aqueous solution containing cellulose@Pt/TiO_2_ (i.e., Pt/TiO_2_ photocatalyst coated by cellulose) [29]. The authors reported a *r*H_2_ = 27 µmol h^−1^ g_cat_^−1^, which despite being lower than that achieved with a 250 W UV lamp (933 µmol h^−1^ g_cat_^−1^), demonstrated the feasibility of using natural sunlight for generating H_2_ from photo-reforming of lignocellulosic materials. It should be noted, however, that the efficiency of photocatalytic reactions (i.e., AQY or STH) under natural sunlight and artificial irradiation is challenging to compare as the light intensity reaching the catalyst, which dictates the photocatalytic activity, could be heavily influenced by photoreactor geometry and engineering. Parameters such as overall configuration, materials, reactor volume, active photocatalyst surface area, mass transport and light distribution are crucial for facilitating an enhanced reaction. Previous studies have considered reactor engineering for photocatalytic H_2_ generation including investigating fluidisation approaches [20, 59], technical and economic feasibility [60] and more recently the deployment of large-scale (100 m^2^) solar photocatalytic water splitting units [61, 62]. To date, however, there have been no reports in the literature which have focussed on reactor design exclusively for photocatalytic biomass reforming.

### Photocatalytic Reforming of Lignin and Lignin Models

To date, the majority of biomass photocatalysis research has focused on cellulose (and/or other carbohydrates) as a model compound where, in addition to the production of H_2_, valuable chemicals can also be formed. The most abundant compounds from cellulose photo-reforming are monosaccharides (such as glucose and arabinose) and HMF [29, 45–48] with cleavage of the β-1,4-glycosidic bond regarded as the key step in the process [30, 65, 66, 69]. Lignin, however, makes up a large portion of raw biomass and is considered the more unreactive and recalcitrant constituent of the native material. Its chemical composition represents a challenge for biomass conversion processes and as a result is often underutilised. Despite this, targeted degradation of lignin has the potential to yield various chemicals of potential interest to the fine chemical industry [70] and which have been produced alongside the production of H_2_ in several systems.

In terms of the formation of both an energy vector and valuable products from lignin-based feedstocks, several studies have been reported in the literature. Zhao et al. performed photo-reforming of Kraft lignin in 1 M NaOH solution with a TiO_2_-NiO n-p heterojunction catalyst to yield both H_2_ and CH_4_ as the gas phase products along with the generation of fatty acids in the liquid phase [49]. These compounds included palmitic (35%), stearic (25%) and butanedioic acid (7%), which are capable of being further converted into biofuels. In this study, the n-p heterojunction of the TiO_2_-NiO catalyst was shown to be effective in separating and migrating the electron–hole pair which facilitated the redox reactions at the catalyst surface. While electrons formed on the TiO_2_ surface led to the formation of H_2_, photogenerated holes facilitated the oxidation of lignin to form the acids [49]. Work conducted by Kadam et al. demonstrated that the photo-reforming of the sodium salt of lignosulfonate using C-, N- and S-doped ZnO catalysts was capable of producing both H_2_ and the primary product 1-phenyl-3-buten-1-ol. The authors proposed H^+^ to be the active species in the photo-oxidation of lignin via direct oxidation at the catalyst surface [68]. Sun et al. [71] studied the photocatalytic cleavage of lignin model compound 2-phenoxy-1-phenylethanol using a Ni/CdS catalyst in various solvents, which were shown to influence the rate and product distribution. The lignin substrate was oxidised to a ketone product (2-phenoxy-1-phenylethanone), which was concurrent with H_2_ generation occurring at the conduction band. The reactions were conducted in pure CH_3_CN and irradiated for a period of 18 h from an 8 W blue LED (wavelength range = 440–460 nm). The authors reported that the selectivity and conversion of the reaction achieved was ~ 100%. In comparison, by using a CH_3_CN/H_2_O (20:80) solution, the study found that the lignin model compound was converted within 8 h of irradiation, and acetophenone and phenol, along with 2-phenoxy-1-phenylethanone, were the photo-oxidation products formed. Furthermore, in alkaline conditions, (i.e. CH_3_CN/0.1 M KOH) full conversion of the lignin substrate was achieved in 3 h of light irradiation with more than 90% acetophenone and phenol formation. It was expected that the H^+^ in Ni/CdS was responsible for the oxidation of the Cα–OH group in 2-phenoxy-1-phenylethanol, resulting in the generation of 2-phenoxy-1-phenylethanone as the primary product. Meanwhile, H_2_ was produced utilising the proton extracted from the 2-phenoxy-1-phenylethanol.

Photo-oxidation for the generation of aromatic compounds from lignins has been more widely studied than the systems described above, which produced both H_2_ and value-added compounds. Selective formation of desired products is however, still a significant challenge for photocatalytic reforming of biomass to chemicals due to unselective oxidation reactions and the complexity of lignin substrates (insolubility and low reactivity). One strategy to improve the interaction of lignin with the catalyst included wet-milling of lignin and TiO_2_ prior to photo-oxidation which resulted in increased depolymerisation to phenolics [72]. An alternative strategy outlined in the study by Wu et al., improved catalyst-substrate interaction through solubilising the catalyst as opposed to lignin. The solubilisation and dispersion of the catalyst in an aqueous methanolic solution, and functionalisation of CdS QDs with 3-mercaptopropionic acid improved the interaction of the catalyst with native lignin [73]. The authors stated that the colloidal CdS QDs were utilised in a ‘lignin-first’ visible light-activated conversion of biomass (birch woodmeal), to monomeric aromatics (~ 27% yield) which proceeded without conversion of cellulose or hemicellulose [73]. Interestingly, following separation of the catalyst, cellulose and hemicellulose fractions could be recovered for acidolysis/enzymatic hydrolysis to form xylose and glucose. The CdS catalyst in this study was reported to activate the dominant linkage in lignin, the β-O-4 bond, which was subsequently cleaved by an electron–hole coupled photoredox mechanism.

**Fig. 5:**
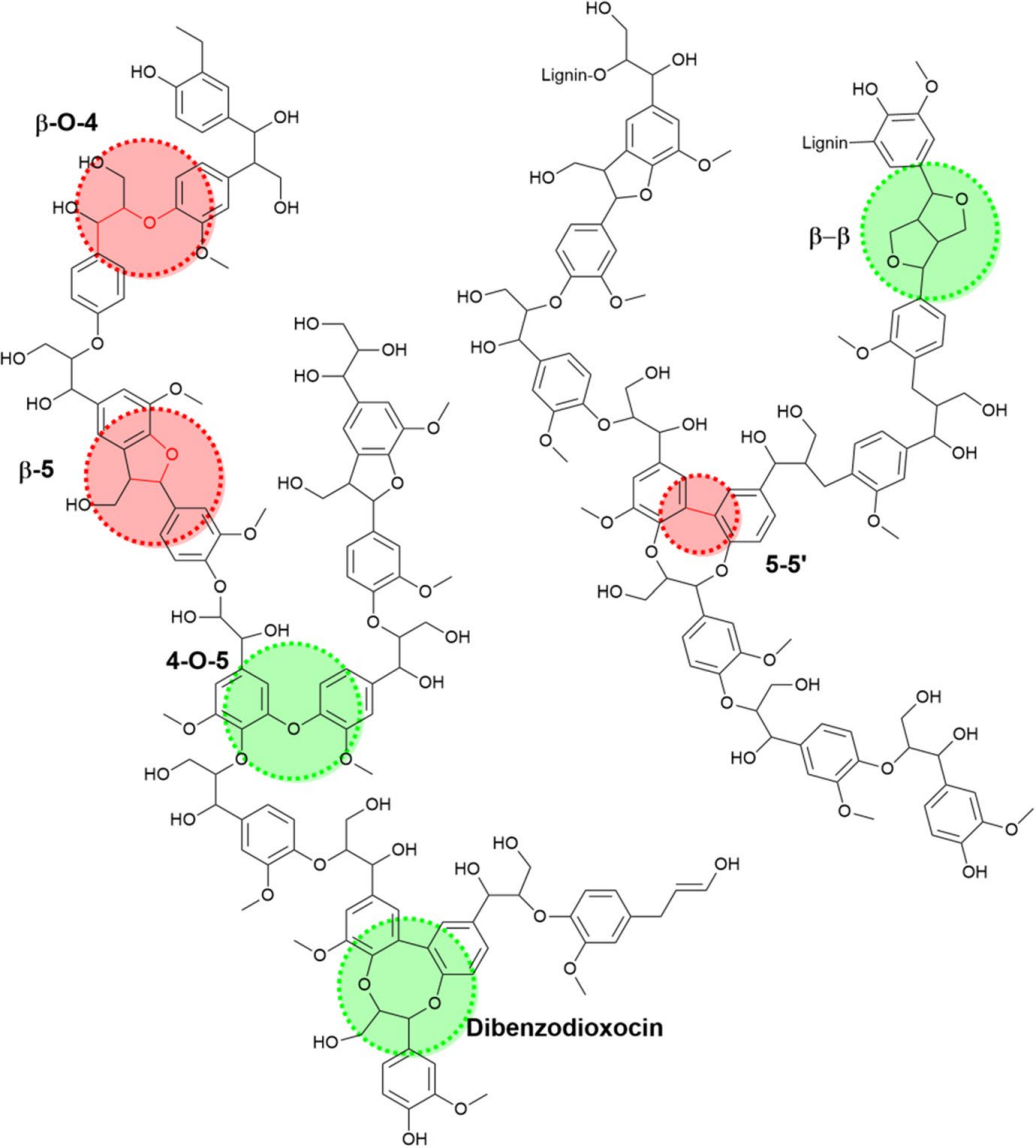
Structure of lignin identifying the key linkages including the β-O-4, β-5 and 5–5’ biphenyl bonding patterns (in red)

While the use of raw biomass and lignins represents a clear step forward for photocatalytic reforming of biomass, it also highlights the need for fundamental research into the effect of the technology on different lignin bonding patterns. As such, there is research which has focused on exploring the impact photocatalysis can have on the lignin structure through deploying model compounds which contain the key linkages found in the native material, e.g., β-O-4 [74–79], β-5 [80] and 5–5’ [81] linkages. The proportion of these bonding patterns varies within lignin; however, in softwood species the relative proportions of β-O-4, β-5 and 5–5’ biphenyl are 45–50, 9–12 and 19–22%, respectively [82]. The structure of lignin is shown in Fig. 5, which highlights the range of bonding patterns present in the material.

**Fig. 6:**
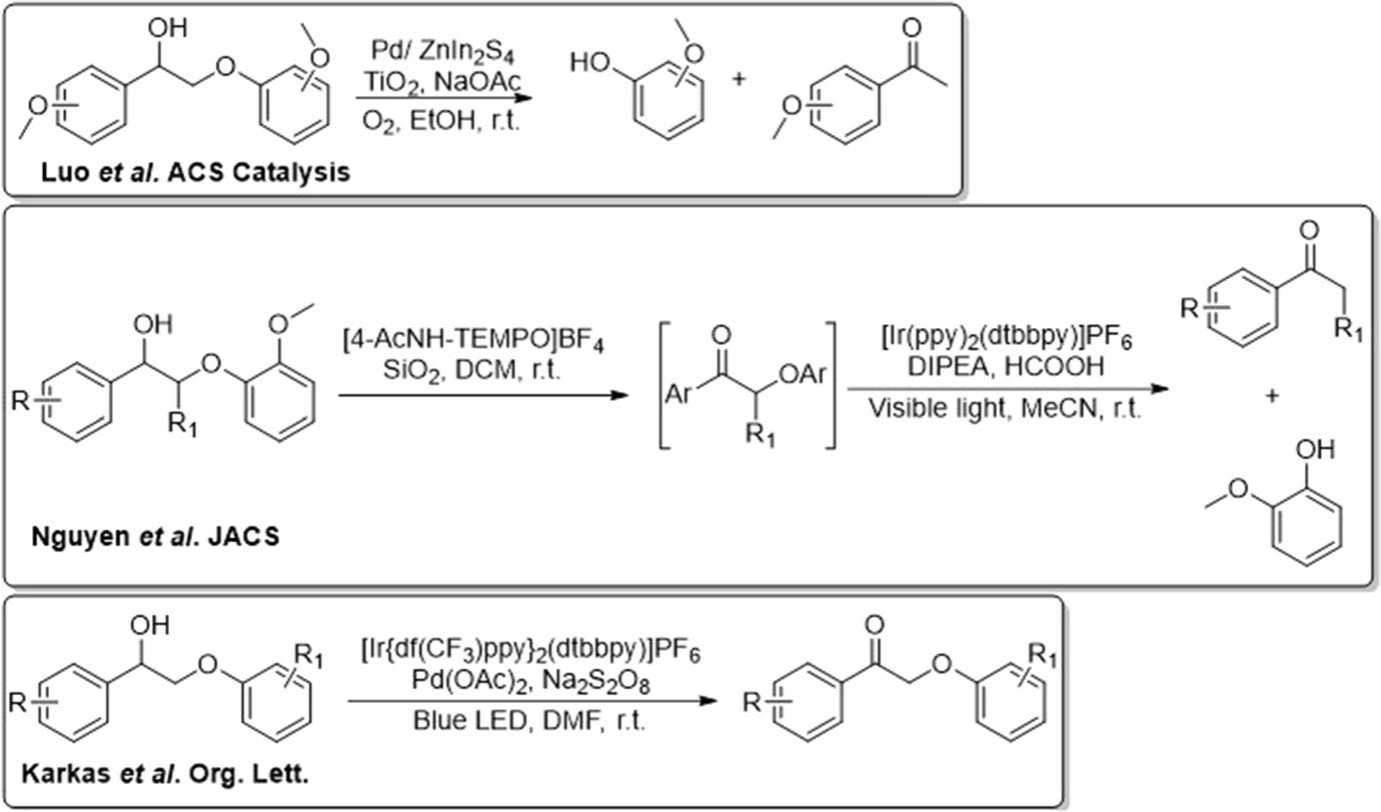
Transformations employed for the conversion of β-O-4 model compounds. Information shown in the figure is reprinted (and adapted) with permission from [76–78]. Copyright 2022, American Chemical Society

Model compounds containing the β-O-4 linkage have been the most reported in the literature, which is because the linkage is the most abundant in native lignin and the relative simplicity of synthesising the materials. An overview of the transformation detailed in the literature is shown in Fig. 6. Work by Stephenson et al. demonstrated that photo-redox catalysis was capable of degrading lignin model compounds containing a β-O-4 linkage [76, 77]. The group developed a dual-catalytic system whereby photo-redox was followed by a co-catalytic system of palladium in the presence of sodium persulfate as a terminal oxidant. An octahedral iridium complex as a photocatalyst along with palladium catalysis could undergo a single electron transfer to perform an α-oxidation thus weakening the adjacent bonds in the linkage. The use of the dual-catalytic strategy allowed for the oxidation of both primary and secondary alcohols within the β-O-4 model compound, followed by fragmentation via C–O and C–C bond cleavage.

A combined approach was also adopted by both Wang et al. [78] and Zhang et al. [83]. The former utilised photocatalytic oxidation with hydrogenolysis in a dual-light wavelength switching strategy, while the latter utilised electrochemical oxidation followed by photocatalytic C-O bond cleavage. The work by Wang et al. demonstrated that β-C–O bonds can be cleaved in β-O-4 model compounds, with the authors proposing that a radical driven process produced two fragments of the β-O-4 model compound. In the work by Zhang et al., the rationale behind this approach was supported by previous calculations by Beckham et al. [84], whereby it was shown that oxidation of a benzylic alcohol (to provide the carbonyl) weakened the β-C-O bond by as much as 13.3 kcal mol^−1^. The dual strategy meant that once this bond was effectively weakened due to the oxidation process, the photocatalytic cleavage of this bond was more favourable.

In contrast to the β-O-4 linkage, research on the photocatalysis of model compounds containing the β-5 and the 5–5’ biphenyl linkages have been limited. While there are several studies detailing syntheses of the compounds [85–88], work on their degradation is not frequently reported. Castellan et al. [89] showed that by using the biphenyl models divanillin and divanillyl alcohol, degradation can be achieved using TiO_2_ photocatalysis in an ethanol–water solvent mixture.

**Fig. 7:**
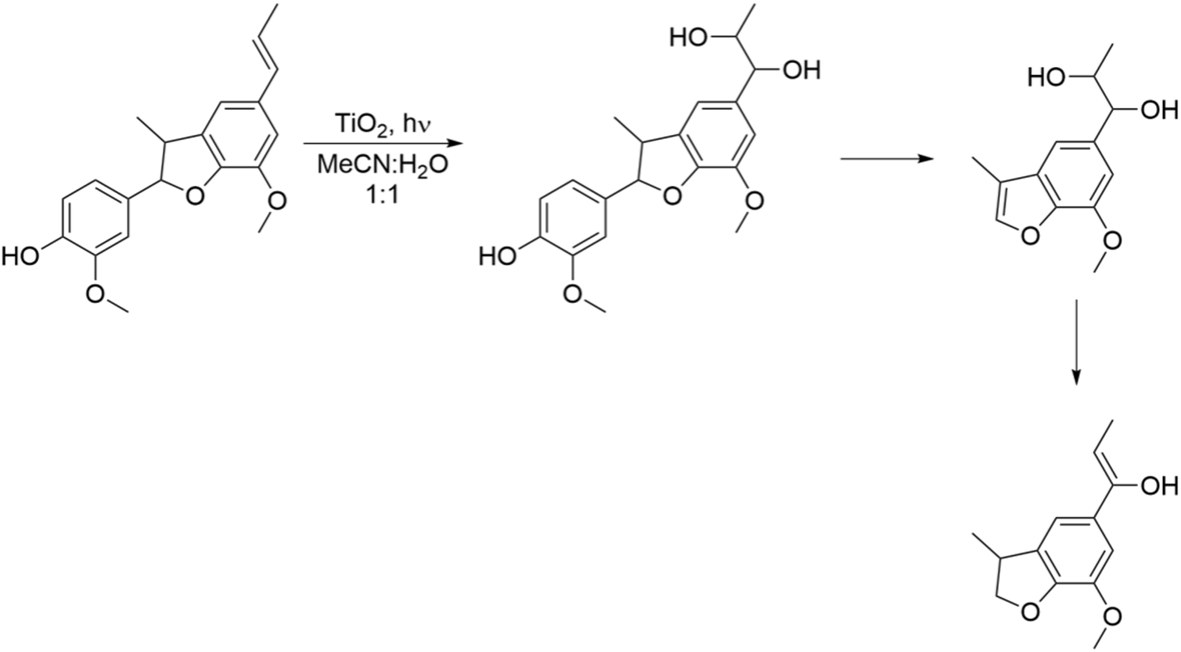
Scheme of events leading to the products found in the TiO_2_-mediated photocatalytic degradation of a β-5 model compound [90]

Similarly, research associated with β-5 linkage has typically focussed on synthesis, as opposed to photocatalytic conversion. Recently, Murnaghan and coworkers demonstrated the use of TiO_2_ in the photocatalytic degradation of a β-5 model dimer [80]. Under low power (~ 1 W) UV-LED irradiation, complete degradation of the β-5 compound was achieved along with formation and subsequent removal of reaction intermediates. Murnaghan et al. [90] observed the generation of a diol species within 2 mins of irradiation which was determined as the initial step in the conversion of the dimer. Subsequently, further bond cleavage resulted in the formation of several key reaction intermediates, Fig. 7.

## Future Perspective for Photocatalytic Reforming of Biomass 

Providing a future perspective on photocatalytic reforming of biomass is challenging, especially considering it is a research topic which is still relatively new. Part of providing that perspective comes from identifying the current technology status along with the target status and reviewing the challenges that separate those two points. Beyond that, however, it is crucial to explore the additional parameters which can influence technology development, and in particular the ‘incentive’ which can drive this, e.g., industrial engagement. This section addresses these points in more detail by adopting a holistic view of photocatalysis as a process and technology for the conversion of biomass to energy.

### Assessing the TRL of Biomass Photocatalysis

**Fig. 8:**
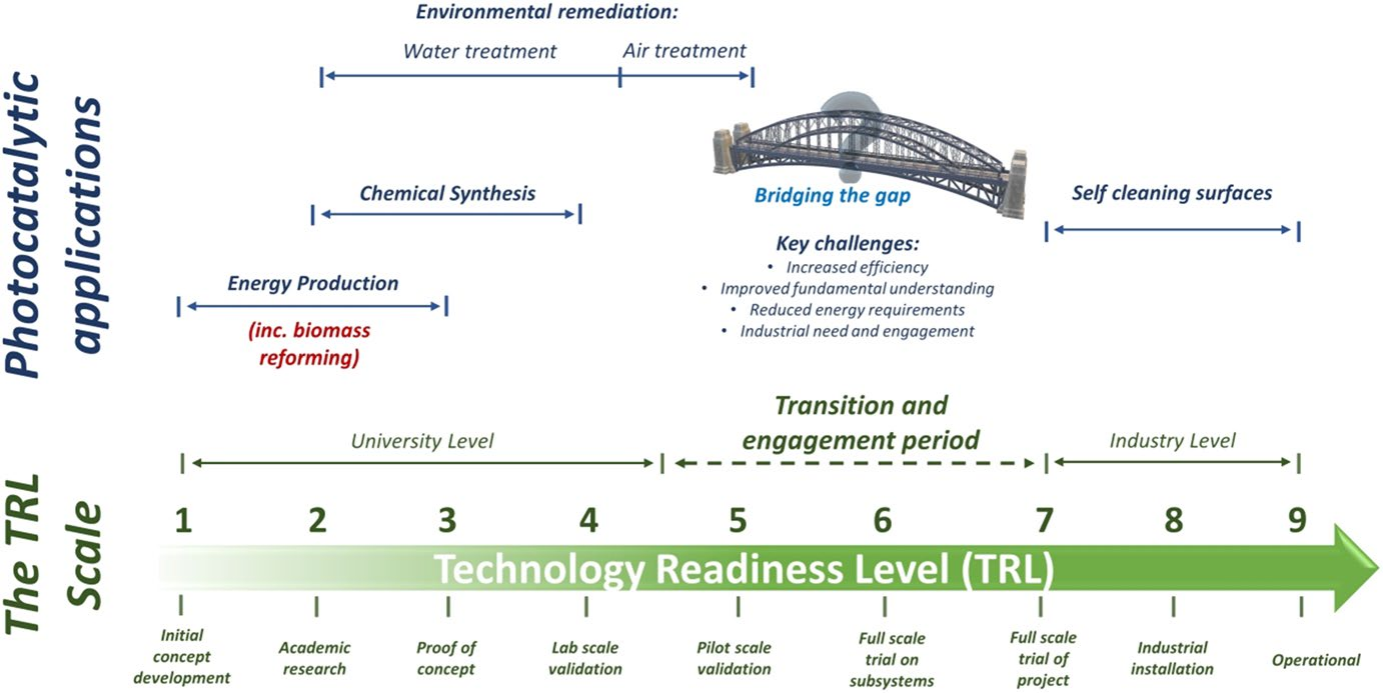
An overview of the TRL scale for photocatalytic applications. Adapted from [34] with kind permission from Elsevier

The use of a simple 1–9 scale, which is accompanied by a clear description for each position, allows the TRL scale to become an extremely valuable tool for assessing emerging technologies such as photocatalysis. The diverse range of applications photocatalysis has been applied to has resulted in the technology covering the entire TRL scale. While a TRL of 9 indicates a mature technology that has been deployed at scale, there are few examples of photocatalytic processes achieving this, with self-cleaning the most notable [91, 92]. In contrast, most applications remain within a TRL range of 1–6 with environmental remediation considered the more developed process when compared to alternatives such as chemical synthesis and energy production, Fig. 8.

**Fig. 9:**
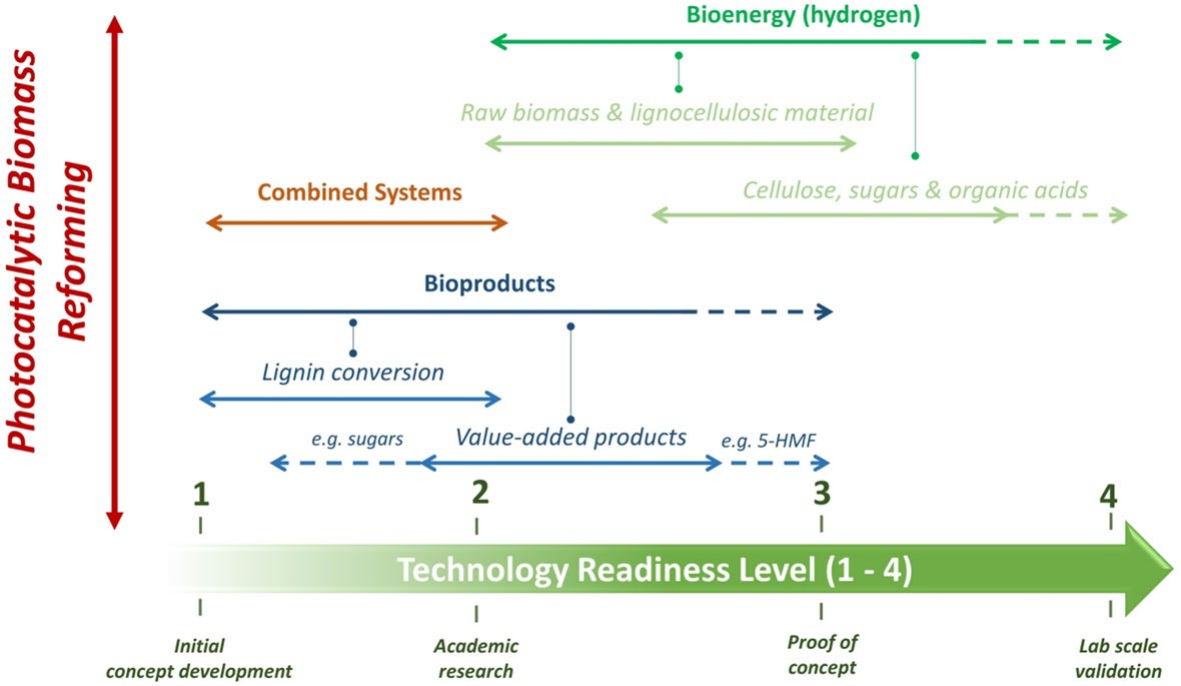
The TRL scale in relation to photocatalytic biomass reforming, where solid lines represent the current range for specific applications and the dashed lines represent potential for expansion (based on recent or related literature)

The TRL position of photocatalytic processes can often reflect the chemical complexity and challenges associated with these applications. The non-selective nature of ROS facilitates the removal of contaminates from air and water more effectively than they do for the generation of an energy vector (e.g., H_2_) or desirable bioproduct (e.g., value-added compounds). Moreover, the presence of competing reactions such as electron scavenging, and reaction product oxidation can further reduce the potential feasibility of the process. Subsequently, the TRL position of photocatalytic reforming of biomass can be seen in Fig. 9, where it is positioned between 1–3. If the primary applications are considered as H_2_ generation, bioproduct formation and combined systems (e.g., simultaneous energy and value-added compounds), additional subcategories can also be included on the TRL scale. A key factor when assessing the TRL status of any technology, is the requirement that the conditions of a certain level (e.g., TRL 5) are achieved or demonstrated for a technology to be considered at that level. For instance, a technology at TRL 5 does not progress to 6 simply by achieving the criteria of 5—it must demonstrate the requirements of TRL 6 first. Therefore, this allows photocatalysis technologies to be viewed in relation to both what has been achieved and what must still be achieved to further develop. As Fig. 9 highlights, H_2_ generation can be considered the more advanced technology within biomass photocatalysis with a TRL of 3. Interestingly, this value can vary if the subsections of feedstock are considered; raw biomass (e.g., lignocellulose and/or only lignin) is still at the proof-of-concept stage (TRL 1–2) while alternative feedstocks (e.g., sugars or organic acids) are supported by more substantial laboratory data and as such are approaching lab scale validation. The latter is likely a result of sugars and organic acids being utilised as SEDs for H_2_ generation for several years prior to photocatalysis being deployed as a method for biomass conversion. Moreover, if biomass is considered as a (more cost effective) SED, photo-reforming to H_2_ can also build on the extensive research conducted into photocatalytic water-splitting and H_2_ generation (from other SEDs). This may also include examples of larger scale solar photocatalytic H_2_ generation, and more recently the solar water splitting photoreactors in Japan [61, 93, 94]

In contrast, bioproduct generation and combined systems have a TRL of 1–2, as research in this area is still in its infancy. As highlighted in the Figure, subsections for these topics include lignin (and lignin model) conversion and value-added product formation, identifying HMF and sugars as example target compounds. A potential limiting factor within this area is selectivity of products and overall yield, which has resulted in literature focusing more on H_2_ generation. It is worth noting, however, that research into value-added compounds can be more focussed on the fundamental chemistry associated with the mechanisms, e.g., the use of lignin models to identify which linkages are cleaved during the reactions. While TRL advancement in this area may be restricted, the work could be highly complementary to enhancing the understanding of how H_2_ is formed, which in turn would facilitate the development of that application towards a higher TRL. The source of protons in biomass photocatalysis processes remains relatively unknown due to the presence of multiple reaction intermediates in the oxidation pathway. An improved understanding of that could be utilised to increase the concentration of protons available at the catalyst surface for accelerated H_2_ production.

**Fig. 10:**
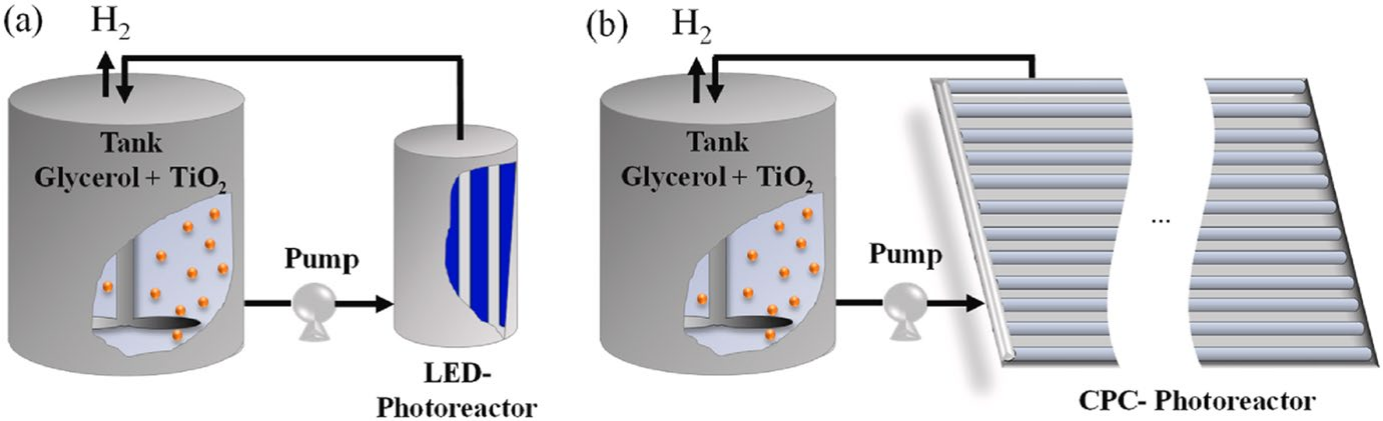
Reactor configurations considered in the work by Rumayor et al. [95] for the photo-reforming of glycerol to H_2_, where (**a**) consists of a LED photoreactor and (**b**) consists of a CPC-photoreactor. Image reprinted with kind permission from Elsevier

**Fig. 11:**
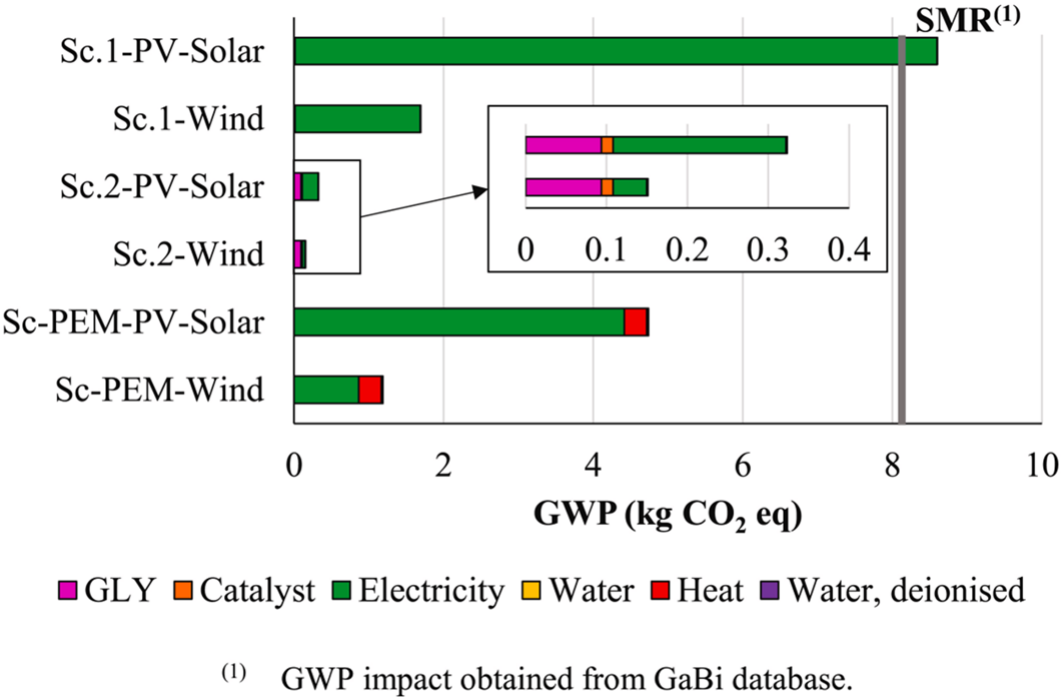
The results of the LCA conducted by Rumayor et al. [95] in relation to Global Warming Potential (GWP) of different photocatalytic glycerol reforming scenarios (Sc) and reference scenarios for polymer electrolyte membrane (PEM) electrolysers. Sc-PEM per functional unit (1 kg of H_2_). Image reprinted with kind permission from Elsevier

Therefore, by adopting a more holistic evaluation of the technology through tools such as the TRL scale, the interdependency of these applications can be demonstrated. Interestingly, there is evidence of this approach now being adopted within recent literature for the application of waste photo-reforming. Rumayor et al. performed a life cycle analysis (LCA) on the photocatalytic reforming of waste (e.g., glycerol) for H_2_ generation with a view towards evaluating the environmental impact and potential feasibility of such a system [95]. Their approach used both water electrolysis and SMR (i.e., green and grey/blue H_2_, respectively) as benchmarks in the study to ascertain the validity of photocatalytic technology for sustainable H_2_ production in the future. The investigation considered two possible reactor configurations based on the method of irradiation: artificial with LEDs and direct solar via a compact parabolic collector (CPC), Fig. 10. The work considered several operational scenarios and while it demonstrated the challenge in conducting this type of research, especially for an emerging technology, it also underpinned the significant potential photocatalysis technology has moving forward. This was demonstrated in relation to global warming potential (GWP), which identified direct solar irradiation (e.g., Sc. 2-PV-Solar and Sc. 2-Wind) as having the lowest potential GWP at < 0.4 kg CO_2_-eq (Fig. 11). Under scenario 2 (Sc. 2) renewable energy was utilised only for mixing and stirring as opposed to Sc. 1 which utilised renewable energy for mixing, stirring and the LED irradiation array. The authors concluded that even under low production rate conditions (e.g., ~ 5 × 10^−4^ kg H_2_ hr^−1^), photocatalytic technology in both scenarios could provide green H_2_ with a GWP that was comparable to other renewable driven processes, e.g., water electrolysis. While that statement alone is a substantial observation within the context of photocatalysis research, it is crucial to consider two additional points. This paper is one of the first to attempt an LCA-based study of photocatalytic H_2_ generation, which is a potential indication of the status the technology is reaching. Secondly, the scenarios presented in the study were, as the authors stated, ‘a simplification of a complex reality’. Therefore, it is paramount that studies such as this one be utilised in conjunction with identifying and addressing the additional challenges which are present in this area.

### Identifying Challenges

The challenges associated with any photocatalytic process can often be viewed under the core headings of catalyst (including the reaction kinetics), reactor, and lights. These can also be applied to photocatalytic biomass conversion; however, it is important to note that there are specific challenges within this topic that are not associated with processes such as water splitting and/or CO_2_ reduction. In evaluating such a system, it is important to understand from the outset what would be regarded as a realistic target for an efficient process. This can be assessed with respect to the nature of the biomass feedstock, what the target products are, what yields are achievable for these products and what the overall efficiency of the process is in achieving these yields.

With respect to the photocatalyst and the reaction (e.g., substrate conversion), the nature of the feedstock will be a critical initial consideration. As discussed in Sect. 2, the photocatalytic reforming of a range of different biomass feedstocks has been reported in the literature ranging from refined cellulose through to grass. Each of these feedstocks presents challenges in terms of effective deployment and distribution of the material relative to the photocatalyst. The presence of biomass as a suspended solid within a photocatalytic reactor limits catalyst-substrate interaction and as a result can inhibit the effectiveness of ROS. This can also be coupled with the recalcitrant nature of biomass which again can present a challenge for ROS, especially if bond cleavage (e.g., lignin linkages and glycosidic bonds) is not achieved. Therefore, depending on the feedstock there may be a requirement for either chemical or physical pre-treatment prior to deployment in a photocatalytic reactor. The inclusion of such a step may influence the overall viability of a scaled system, much in the same way as filtration (or additional downstream processing) does for a TiO_2_ slurry reactor. The financial and energy costs of such processes must be considered when assessing the overall efficiency. A final consideration for biomass conversion technologies is the source (i.e., imported, nationally distributed or locally sourced) and availability (i.e., quantity and consistency of supply) of the biomass feedstock itself. There is little point in developing a system for biomass resources which are in limited supply or varied purity and/or form which would necessitate re-engineering of the reactor and catalyst setup to ensure a practical product yield.

Additional points related to the photocatalyst include both the yield of product generated and under what irradiation source. As previously highlighted, the product yields of both H_2_ and chemicals, such as carbohydrates, generated from the photocatalytic valorisation of biomass are still low—typically in the micromolar scale after several hours of irradiation. This is significantly lower than the litre or cubic metre scale that would be required for a practical system even at a prototype level. The challenge in increasing the scale of product yields and efficiency of the process will depend on several factors, particularly the photocatalyst material itself. This is further influenced by the electronic structure of the catalyst and its potential for visible (or solar) light activation. Due to the lower attenuation of visible light compared to UV through fluids and also the potential for utilising solar light, a visible-light active photocatalyst would be highly desirable for photocatalytic valorisation of biomass. Since the late 1990s there have been extensive reports of visible-light active photocatalyst material for both water treatment and energy conversion applications, which have often had variable efficiencies. Even if an effective photocatalyst material which can generate molar quantities of products (or even cubic metre levels per hour) was generated, that material will need to be viable for commercial scale production. With the exception of a modified TiO_2_ material, produced by Kronos, no visible light-active materials are currently available commercially. For any process to be practical on a large scale, photocatalyst materials will need to be available in kilogram/tonne quantities. Consequently, for any potential upscaling, at the moment, a commercially available photocatalytic material (such as Evonik TiO_2_) would need to be utilised as the photocatalyst. While this material is available in bulk quantities, it still requires UV light (~ 385 nm) to activate it.

Moving beyond the challenges of catalyst development, the method of deployment becomes paramount. This includes the role the reactor plays in maximising the active photocatalyst surface area, light intensity and distribution, mass transport and product separation. In terms of the scope of this article, it is not possible to go into all the specific engineering challenges in depth, and to do so would be the subject of a detailed article in itself. The issues raised here, however, are some of the key issues that would be considered the most significant challenges for a biomass-based system and those that would need to be addressed in developing a prototype or small-scale units before considering larger scale systems. The reader is directed towards several excellent papers that review and investigate the specific details of photoreactor concepts [96–100].

Within photoreactor design, reduced mass transfer and enhanced light distribution are two parameters which present a challenge when considering biomass feedstocks. Ensuring effective mass transport in photocatalytic reactors is often challenging, and this is further increased in the conversion of biomass due to the recalcitrant and insoluble nature of the material. Moreover, the presence of suspended biomass particles within both flow and batch reactors can also impact the rate of conversion. Therefore, providing an active catalyst is deployed (as either a suspension or immobilised onto a support), it is crucial the reactor can maximise the interaction between the suspended feedstock and catalyst surface. While this point has not been exclusively addressed in the literature for this topic, it has been an issue when investigating reactor engineering characteristics for other photocatalytic processes [101–104]. Several approaches have been previously adopted in photocatalytic treatments systems including the use of enhanced flow systems and agitation via mixing or sparging with air/oxygen, application of rotating disc reactors and use of turbulence promoters such as baffles [20, 105–108].

In addition to mass transfer limitations, effective distribution of photons within a photoreactor becomes challenging because biomass feedstocks are capable of absorbing and scattering light. Moreover, the turbidity created from a biomass suspension can also reduce light penetration and subsequently limit photon absorption at the catalyst surface. Even when the method for determining irradiation intensity in a photocatalytic reactor is clear, how the light is distributed within that reactor and how much is actually involved in the process is not always apparent. Understanding this, however, is crucial as it provides a platform to then assess the potential impact of biomass within the reactor. Meng et al. (2019) discussed how the radiation transport equation (RTE) can be used to model light intensity distribution on photocatalytic reactors [109]. The light intensity as a stream of photons that moves through the photocatalytic reactor will depend on the following parameters: absorption of light, out-scattering of light and in-scattering of light. The out-scattering of light occurs when light is scattered out of the light path and is, therefore, deemed not to be available for interaction with photoactive materials. In contrast, in-scattering refers to light that is scattered back into the light path and hence is available for interaction with photoactive materials. The RTE for a photocatalytic reactor is a combination of the absorption, out-scattering and in-scattering terms (Eq. 1) [109].

1 $$\frac{{dI_{\upsilon } (s,\Omega )}}{{ds}} = \underbrace {{ - \kappa _{\upsilon } I_{\upsilon } (s,\Omega )}}_{{{\text{Absorbed light}}}} - \underbrace {{\sigma _{\upsilon } I_{\upsilon } (s,\Omega )}}_{{{\text{out - scattered light}}}} + \underbrace {{\frac{1}{{4\pi }}\sigma _{\upsilon } \int\limits_{{4\pi }} {p(\Omega ^{\prime} \to \Omega )I_{p} (s,\Omega ^{\prime})d\Omega ^{\prime}} }}_{{{\text{In - scattered light}}}},$$

where I_υ_ (s,Ω) is the monochromatic light intensity along the path s in the direction Ω, k_υ_ is the coefficient of absorption, σ_υ_ is the coefficient of out-scattering, Ω is the direction of the light stream, Ω’ is the direction of light from other directions and p(Ω’ →  Ω) is the phase function of in-scattered incident irradiation to an element from other directions (Ω’) [109].

This equation highlights the complication with biomass photocatalysis as the material itself is likely to both absorb and scatter light and hence limit the intensity reaching the surface of the photocatalyst material. This will clearly have an impact on overall efficiency of the process but in particular the quantum/photonic efficiency. Under such conditions, ensuring that light can be effectively delivered to the surface of the photocatalyst in the presence of a complex biomass material is not simple. As a result, this becomes a challenge that must be addressed by reactor engineering in view of the geometry between catalyst deployment and irradiation source array. The immobilisation of the photocatalyst to a substrate which allows direct catalyst irradiation (e.g., irradiation through a transparent support or via an optical fibre coated with a photocatalyst) rather than through the biomass matrix could aid this process. The limitation to this approach, however, is that the relative active photocatalyst surface area will be reduced if compared to that achieved for a suspended catalyst system with a similar footprint.

Finally, an important aspect of developing photocatalytic systems for biomass conversion is the metric which is used to assess activity. Assuming an active catalyst is effectively deployed in a suitable reactor which can utilise a range of biomass-based feedstocks, the metric by which that activity is measured becomes a crucial consideration. As with other photocatalytic processes, selection of an appropriate measure of efficiency is an issue, as there is often inconsistency in how this is reported between different laboratories. Many groups report efficiencies of processes in terms of yields of products by the photocatalyst (e.g., µmol h^−1^g_cat_^−1^), while others report the quantum (or formal quantum) efficiencies based on the ratio between the reaction rate and number of photons (which is often represented as an intensity). While such measures are appropriate for photochemical/catalytic processes, they are less informative for more whole-system comparisons. This point also highlights another key challenge within photocatalysis which is associated with the lack of standardised testing to provide a more accurate platform for comparison. The use of an appropriate metric could be extremely valuable for encouraging engagement with potential industry stakeholders. While a photonic efficiency is useful at lab scale, it offers a limited insight into the performance of the entire system, which is what would be required to demonstrate pilot scale feasibility.

### Roadmap for Technology Deployment

The previous sections highlighted the technology status of photocatalytic reforming of biomass along with the challenges associated with advancing the field. While these challenges are significant and require dedicated research to address and potentially overcome them, the deployment strategy for photocatalytic technology can also influence the research conducted with a view towards establishing a roadmap. The literature has often stated the main objective for photocatalysis as being towards industry level operation and deployment, and while that will always remain as the core objective, examples of this being achieved are very limited. This point was also discussed in the excellent work by Loeb et al. when they reviewed the technology horizon for photocatalytic water treatment, specifically identifying that ‘technology transfer problems’ are being overlooked which widens the gap between research and industry [110]. As discussed by Loeb and colleagues in their feature article, this issue is somewhat reflected in literature trends; of the 1356 papers published on photocatalytic water treatment in 2017, only 29 contained the term ‘pilot’. Given the TRL status of that application, a higher proportion of industry-facing research would be expected. Their literature review, however, suggests topics such as reactor design and pilot-scale studies are not as routinely investigated as those on reaction kinetics and material synthesis.

**Fig. 12:**
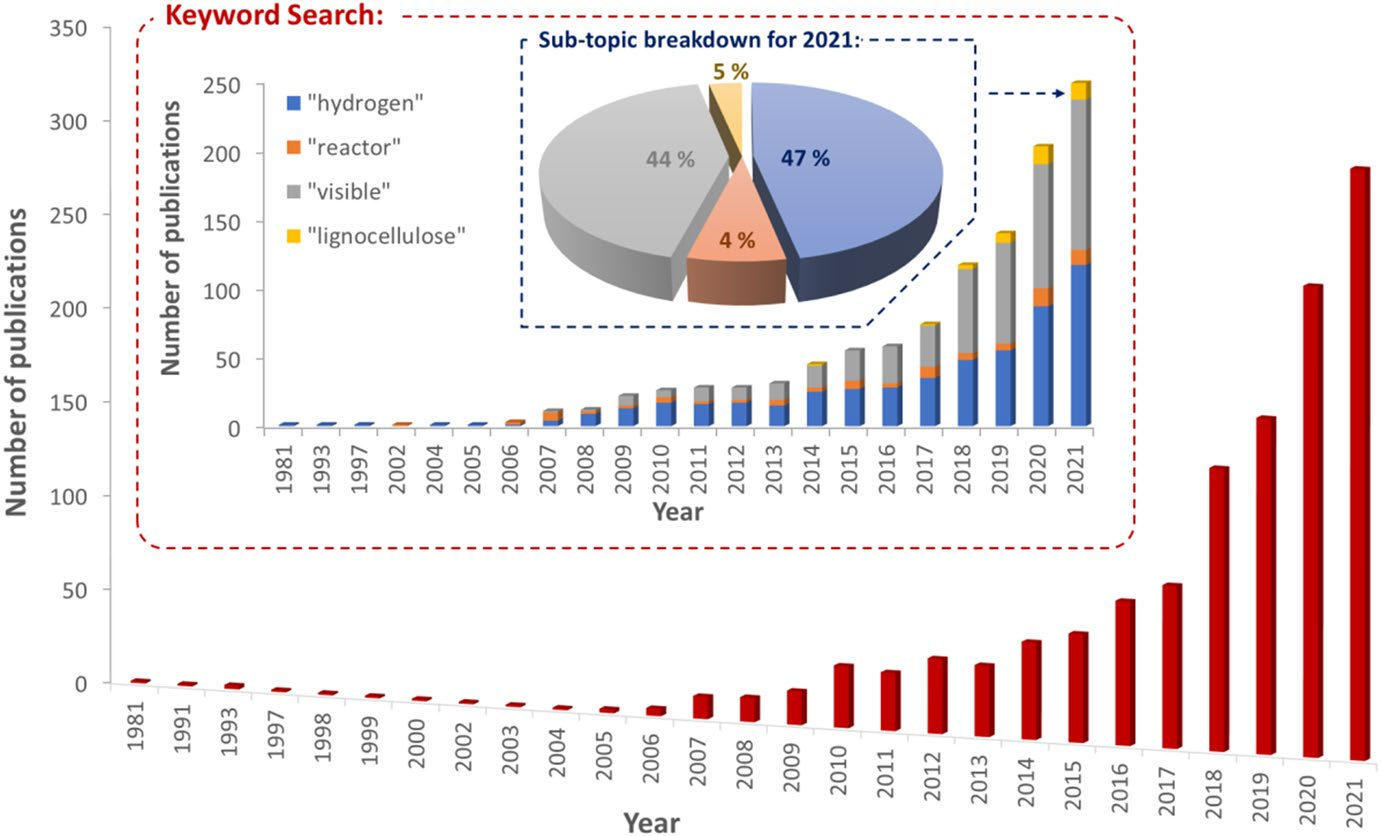
Literature trends for publications on photocatalysis biomass research. The main graph shows the number of publications from a Web of Science search using the keywords ‘photocatal*’ and ‘biomass’ during 1981–2021. The Keyword Search insert provides a further breakdown of the literature trends during the same period based on additional keywords of ‘hydrogen’, ‘reactor’, ‘visible’ and ‘lignocellulose’, while the sub-topic breakdown for the 2021 insert shows the percentage of papers published in 2021 that contained those same keywords

In comparison to photocatalytic biomass conversion, the issues surrounding technology transfer barriers are further emphasised, primarily due to the maturity of the application. With that being said, there is justification for the argument that the research in this field is simply at too early a stage to be considering a roadmap or deployment strategy. There is, however, evidence in recent literature that highlights a potential transition towards process design for solar-to-H_2_ technologies being more prominent. In 2021, the largest to-date solar H_2_ production unit of 100 m^2^ was reported by Nishiyama et al., which was composed of panel reactor arrays with SrTiO_3_:Al as the photocatalyst [61]. In addition, the rapid growth of this field is demonstrated by Fig. 12, which also highlights the research trends emerging within the literature. Despite the first publication on this topic being in 1981, it was not until 2010 that a more substantial increase in the number of publications was observed. This was subsequently followed by a significant increase from 2014 onwards, with 315 papers published last year (2021) which contained the keywords ‘photocatal*’ and ‘biomass’. Interestingly, if the literature search is extended to include sub-topics associated with the field, as shown in the Fig. 12 insert, then ‘hydrogen’ and ‘visible’ are clearly the most active research areas accounting for ~ 47 and 44% of the total, respectively (in 2021). It should be noted that the number of publications including the term ‘lignocellulose’ has also increased since 2017 suggesting that the use of raw biomass as a substrate is increasing. Figure 12 also shows that despite an increase, reactor-focussed research remains less prominent than other sub-topics within this field, accounting for only 4% of the total. In relation to addressing potential technology transfer barriers, this may pose a challenge as research continues to focus and develop around materials and not entire systems. These topics are not independent of course, and it is to be expected that, at this stage, research will (and needs to) focus on catalyst enhancement for use with raw biomass. It is crucial, however, that there is an awareness of how these more-active research areas can encourage and facilitate the growth of associated topics such as reactor engineering and whole-systems analysis.

As indicated by the previous TRL scale (Fig. 8) and by Loeb et al. [110], photocatalysis has been shown to struggle when attempting to overcome technology barriers such as pilot-scale validation, large-scale trials and industrial engagement. There can be several contributing factors to this; however, a lack of clearly identified research priorities could be crucial. In the work by Loeb et al., a series of recommendations were made that provided a framework for advancing the field of photocatalytic water treatment towards higher TRL status and integration into existing industry infrastructure. A similar approach can be applied here, despite the application of photocatalytic biomass conversion being at a lower TRL than water treatment, TRL = 3 and 6, respectively, for biomass reforming and environmental remediation. In doing so, it could allow for research priorities to be identified sooner which would ensure that technology transfer barriers are considered alongside fundamental research as opposed to during later stages. Furthermore, there is additional incentive for technology development that is not often considered or associated within other photocatalytic applications. NZE targets and with it, renewable energy strategies, have significantly altered the future energy landscape and rapidly put focus on a range of current and emerging methods that can contribute towards both. While this is applicable to multiple photocatalytic applications, topics such as biomass (and/or waste) conversion and CO_2_ reduction are at the forefront.

Therefore, what does a roadmap for technology development in photocatalytic biomass conversion look like? Answering this question is subject to several factors; however, it can be aided by considering a potential future deployment scenario for photocatalytic biomass conversion technology to produce H_2_. In any biomass conversion technology, the scale of the production and utilisation of H_2_ as an energy vector (or as a chemical feedstock itself), requires consideration in development of a process. For any biomass-to-H_2_ technology, the production/utilisation of H_2_ could be considered as either:

1. a centralised biomass-to-H_2_ process with distribution of the H_2_, for example, integration into the current natural gas infrastructure or
2. as a localised production process with the biomass resource available on the same site as H_2_ production and utilisation.

In any biomass-to-H_2_ process, the availability of the biomass at the scale required for the process is a key consideration. The potentially large scale of H_2_ production required in a centralised–distributed deployment scenario could see blue H_2_ dominate within this strategy in the short to medium term as the feasibility of green H_2_ production from electrolytic water splitting is investigated, for example, through pilot H_2_-to-homes schemes such as Fife H100 [111]. It is, however, within a localised production scenario, where emerging renewable driven H_2_ technologies could be envisaged to play a more significant role.

**Fig. 13:**
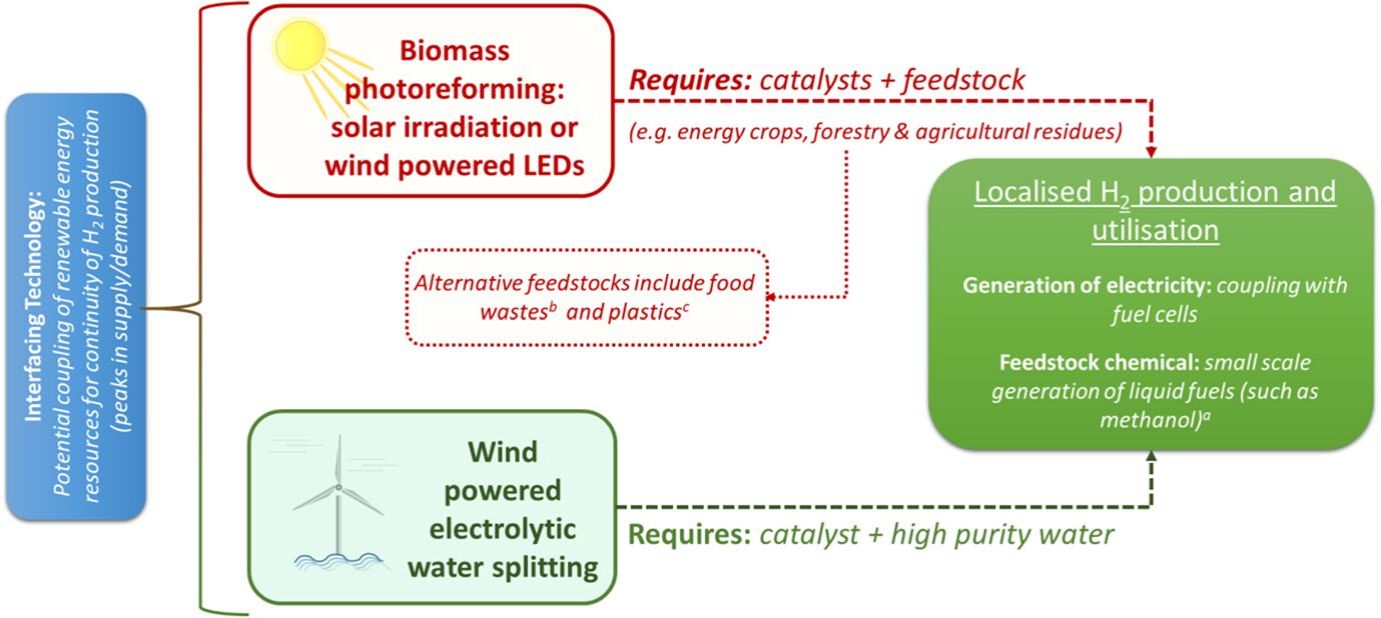
Illustration of a potential deployment scenario for photocatalytic biomass reforming based on a range of feedstocks for H_2_ production and utilisation in electricity generation and/or as a feedstock chemical. References within the figure include [112]^a^, [113]^b^ and.[114]^c^

In the scenario of photo-reforming of biomass for the localised production of H_2_, whilst removal of the biomass transportation costs would provide financial and environmental benefits, the scale of the H_2_ production would still need to reflect the availability of the biomass resource. A scenario could be envisaged where biomass feedstocks, such as energy crops (miscanthus or willow) or wastes from agriculture/forestry could be used in a photo-reforming process activated by sunlight and/or wind-powered LEDs, Fig. 13. The generated H_2_ could be a feed for fuel cells generating electricity which could be used on site (remote locations) or connected to the national grid. Such a scenario requires consideration of the scale and technical specifications of the respective feedstocks for H_2_ production (biomass/water) and utilisation (gas purity/separation) processes but direct use of the H_2_ produced could remove H_2_ storage requirements providing further (financial) advantages for small-scale localised applications.

It is worth noting that the H_2_ (produced from photo-reforming) could be used as a feedstock for production of fuels such as methanol or ammonia or coupled with emerging mild catalytic processes (not requiring pressurised H_2_), e.g., the non-thermal plasma catalytic hydrogenation of CO_2_ and reverse water gas shift under ambient conditions [115–117]. If the H_2_ consumption rate by the catalytic system (fuel cell/plasma reactor) can be matched by the H_2_ production rate from a photo-reforming process, the renewable H_2_ produced can be used locally and immediately for production of electricity or relevant platform chemicals such as CH_4_ and CO.

Perhaps a logical question in relation to the above discussion is what would a proposed reactor design be for such a deployment scenario? Literature on reactor design for photocatalytic H_2_ generation, especially utilising biomass, is scarce, however, which presents a challenge when attempting to forecast what would be required. Currently, the most viable approach would be to recommend desirable design parameters that should be researched in tandem with the design and optimisation of catalysts. As such, two key factors are irradiation, including delivery and distribution, and catalyst deployment which considers immobilised and suspended systems. As discussed, utilising solar energy remains the primary target for irradiation, and where possible reactors should enhance distribution within units to maximise photon absorption at the catalyst surface. Where solar is not suitable, artificial arrays comprising LEDs are highly desirable, providing they are coupled with renewable energy such as wind. Catalyst deployment, however, is a more significant challenge that covers both chemistry and engineering. A catalyst immobilised onto a stationary support can alleviate the downstream processing requirements often associated with suspended systems. A drawback, however, is overcoming the mass transfer limitations which could be significant in a biomass-based photocatalytic system as the feedstock is likely to have low solubility. While not biomass-focussed, two different photocatalytic reactor systems for H_2_ generation from water splitting, namely particle suspensions and planar arrays, were designed and compared by Pinaud et al. [60]. The particle suspension system was an enclosed aqueous reactor bed of suspended photoactive particles, while the system of planar arrays consisted of multilayer absorber planar arrays immersed in an aqueous electrolyte and oriented toward the sun. To compare them, the STH conversion efficiency of each system was estimated, and the results showed that the STH efficiency of single and dual bed particle suspension systems was 10 and 5%, respectively, while the value was 10 and 15% for fixed panel array and tracking concentrator array systems, respectively.

In conjunction with considering a future deployment scenario and reactor design for photocatalytic biomass conversion, the metric by which it is assessed is also crucial. If the focus of biomass conversion is on H_2_ as a vector (and in lieu of a standardised test) an energy balance approach offers a more appropriate route to determining a system efficiency. Put simply, if more energy is being input into the system than is produced by the H_2_ itself, the process is unlikely to be viable in future energy systems. Sathre et al. developed an interesting approach for considering energy balance and life cycle for larger scale photoelectrochemical systems [118]. This elegant modelling approach focussed on three factors:

1. Life cycle primary energy balance
2. Energy return on energy invested
3. Energy payback time.

The life cycle primary energy balance is a measure of the energy provided by the process or system during its lifetime and is calculated using Eq. 2 [118]:

2 $${\text{Energy Balance }} = \, \left[ {{\text{T}}x{\text{E}}_{{\text{H}}} } \right] \, - \, \left[ {{\text{E}}_{{\text{P}}} + \, \left( {{\text{T}}x{\text{E}}_{{\text{O}}} } \right) \, + {\text{ E}}_{{\text{D}}} } \right]$$

where, T is the life of the process/system in years, E_H_ is the energy in H_2_ produced per year, E_P_ is the energy required to produce the process/system, E_O_ is the energy required to operate the system per year and E_D_ is the energy required to decommission the system.

The energy return on energy invested (EROEI) is a measure of the energy that is provided by the system relative to the energy that is input to the system (Eq. 3) [118].

3 $${\text{EROI }} = \frac{{\left[ {{\text{T}}x{\text{E}}_{{\text{H}}} } \right]}}{{\left[ {{\text{E}}_{{\text{P}}} + \, \left( {{\text{T}}x{\text{E}}_{{\text{O}}} } \right) \, + {\text{ E}}_{{\text{D}}} } \right]}}$$

The energy payback time (Eq. 4) [118] considers all energy input and output costs to the system including those associated with decommissioning. This is a measure of the time required for a system to be operated before sufficient H_2_ is generated that will balance the energy that was used in both setting up and decommissioning the plant.

4 $${\text{Energy payback time }} = \frac{{\left[ {{\text{E}}_{{\text{P}}} + {\text{E}}_{{\text{D}}} } \right]}}{{\left[ {{\text{E}}_{{\text{H}}} - {\text{ E}}_{{\text{D}}} } \right]}}$$

The approach developed by Sathre et al. could be adopted as a measure of efficiency of the H_2_ generation from photocatalytic valorisation of biomass and would give a more appropriate method of evaluating the practical viability of the process a pilot/prototype scale and above [118].

### Strategy for Achieving Deployment

Considering both the current start of the literature and the perspective provided in this article for photocatalytic biomass reforming, the authors have suggested the following points as key for developing a strategy for achieving future deployment:

1. ***Catalyst development which facilities technology growth and not academic hype.*** Activity under visible and solar irradiation will remain a primary objective for any photocatalytic application; however, it must be ascertained with due consideration towards additional key parameters. This includes economic and commercial feasibility for large-scale synthesis and deployment, with potential for operation as a thin film or attached to solid support structure. In relation to biomass conversion specifically, the catalyst must be capable of producing a H_2_ yield in a millimole range and demonstrate a photonic efficiency of > 50%. Furthermore, a STH efficiency of 5–10% is recommended to realise economically viable solar H_2_ production.
2. ***Reactor engineering must be elevated to match other key research topics.*** While the design of photocatalytic energy-producing systems remains limited, challenges must be addressed now, regardless of the state of catalyst development. These include using existing examples of modular reactor systems (e.g. electrolysers and fuel cells) to develop modular photocatalytic units which can demonstrate deployment and operation at a range of scales. This must be done with a view towards developing units capable of operating at pilot scale capacity (e.g. 1–5 m^3^). Alongside this, research on irradiation arrays must move away from lab standards (i.e., Xe arc lamps) and focus on its own twin track approach which includes solar and sustainable artificial irradiation. The former does not simply imply that catalysts are placed under direct solar but instead, that systems are engineered which optimise the distribution, delivery and, where necessary, concentration of solar photons to a catalyst surface. The latter should be centred on the development of LED systems which are capable of utilising low-carbon renewable energy such as wind. Finally, consideration should also be given to how such a system can integrate with both existing infrastructure and/or technology for the delivery of H_2_ as an energy vector.
3. ***Establish metrics which encourage engagement at both ends of the TRL scale.*** The two previous points are significantly enhanced by the development and utilisation of accurate and representative metrics as evaluation tools. While a single value or metric is desirable for such a purpose, it often oversimplifies a complex process or fails to consider all key parameters. As such, a combination of metrics must be adopted by research which provides a more substantial evaluation of a system. In the first instance, this can continue to be a *r*H_2_ coupled with a photonic efficiency; however, a modified photocatalytic space time yield [119] should also be incorporated, which integrates reaction rates with reactor engineering parameters such as scale and electrical power. Beyond this, metrics must be focused on whole-systems analysis and specifically for photocatalytic reforming of biomass, that includes an energy balance and life cycle approach (as detailed in Eqs. 2–4). Moreover, with a view towards H_2_ generation, cost analysis (i.e., $/kg H_2_) and environmental impact (i.e., CO_2_-eq/kg H_2_) would provide an indication of performance that is relatable and comparable to current industry standards. It is also crucial that research is conducted to identify these key parameters and more importantly, identify the data sets that are required which allows their determination, e.g., LCA.
4. ***A more even distribution of research priorities and themes is now required.*** While catalyst development will always be a prominent area of research for photocatalysis, it must now make way for more industry-facing topics and themes to improve the overall TRL. This includes the topics discussed in the previous points such as reactor engineering, metric development, and catalyst synthesis and subsequently related topics such as standardised testing. Specifically for photocatalytic reforming of biomass; however, a key research priority is whole-systems analysis through techno-economic analysis (TEA) and LCA. The justification for this is twofold on account of TEA and LCA being able to evaluate and forecast research hot spots. The evaluation of an entire photocatalytic biomass reforming system is extremely valuable in relation to providing accurate metrics which facilitates academic-industrial engagement. Moreover, this approach can also identify where further information is required and, to an extent, identify research priorities. This would include developing an improved life cycle inventory (LCI) for photocatalytic reforming of biomass which would maximise the output of the system analysis.

## Conclusions

Analysing the literature published on photocatalytic reforming of biomass highlights both the potential of the field and how far the technology still must go to achieve impact. The challenges associated with the latter, however, should not force this application into another academic hype cycle which hinders technology advancement. In contrast, progress to date can be used as a platform to identify key research priorities that can be incorporated into developing a technology roadmap and deployment strategy for photocatalytic biomass reforming. These should include catalyst synthesis for materials that are both solar activated and economically viable for commercial production. In addition, reactor engineering must focus on the design of systems which enhance biomass substrate–catalyst interactions while promoting potential pilot scale deployment. Beyond this, whole-systems analysis (i.e., LCA and TEA) and technology integration (i.e., wind-powered LED arrays) must come to the forefront of photocatalysis research. Moreover, it is crucial that this is accompanied by accurate and representative metrics that act as evaluation tools to enhance stakeholder engagement across academia and industry. In doing so, the TRL of photocatalytic reforming of biomass can increase and with it, the potential for the technology to be deployed in future energy systems capable of achieving net zero emission targets.
